# Sorbicillinoid Derivatives with the Radical Scavenging Activities from the Marine-Derived Fungus *Acremonium chrysogenum* C10

**DOI:** 10.3390/jof8050530

**Published:** 2022-05-20

**Authors:** Chengbao Duan, Shiyuan Wang, Ruiyun Huo, Erwei Li, Min Wang, Jinwei Ren, Yuanyuan Pan, Ling Liu, Gang Liu

**Affiliations:** 1 State Key Laboratory of Mycology, Institute of Microbiology, Chinese Academy of Sciences, Beijing 100101, China; 18813111804@163.com (C.D.); wangshiyuan20@mails.ucas.ac.cn (S.W.); huory@im.ac.cn (R.H.); renjw@im.ac.cn (J.R.); 2 College of Life Sciences, University of Chinese Academy of Sciences, Beijing 100049, China; 3 China Institutional Center for Shared Technologies and Facilities, Institute of Microbiology, Chinese Academy of Sciences, Beijing 100101, China; liew@im.ac.cn; 4 State Key Laboratory of Microbial Resources, Institute of Microbiology, Chinese Academy of Sciences, Beijing 100101, China; hualuozhisheng@126.com

**Keywords:** sorbicillinoids, *Acremonium chrysogenum*, structure elucidation, DPPH radical scavenging activity, antimicrobial compounds, marine natural products

## Abstract

Sorbicillinoids are a class of structurally diverse hexaketide metabolites with good biological activities. To explore new structural sorbicillinoids and their bioactivities, the marine-derived fungus *Acremonium chrysogenum* C10 was studied. Three new sorbicillinoid derivatives, acresorbicillinols A–C (**1**–**3**), along with five known ones, trichotetronine (**4**), trichodimerol (**5**), demethyltrichodimerol (**6**), trichopyrone (**7**) and oxosorbicillinol (**8**), were isolated. The structures of new sorbicillinoids were elucidated by analysis of nuclear magnetic resonance (NMR) and high-resolution electrospray ionization mass spectroscopy (HRESIMS). The absolute configurations of compounds **1**–**3** were determined by comparison of the experimental and calculated electronic circular dichroism (ECD) spectra. Compound **3** exhibited a strong 2,2-diphenyl-1-picrylhydrazyl (DPPH) radical scavenging activity, with the IC_50_ value ranging from 11.53 ± 1.53 to 60.29 ± 6.28 μM in 24 h. Additionally, compounds **2** and **3** showed moderate activities against *Staphylococcus aureus* and *Cryptococcus neoformans*, with IC_50_ values of 86.93 ± 1.72 and 69.06 ± 10.50 μM, respectively. The boundary of sorbicillinoid biosynthetic gene cluster in *A. chrysogenum* was confirmed by transcriptional analysis, and the biosynthetic pathway of compounds **1**–**8** was also proposed. In summary, our results indicated that *A. chrysogenum* is an important reservoir of sorbicillinoid derivatives, and compound **3** has the potential for new natural agents in DPPH radical scavenging.

## 1. Introduction

Marine-derived fungi can thrive in the extreme environments including salinity, high pressure, low temperature and oligotrophic conditions compared to their terrestrial coun-terparts, which makes them able to produce structurally diverse bioactive compounds more easily [1,2,3]. Meanwhile, these compounds usually have unique structures that also provide the possibility for structural design and modification of the leading compounds [4]. As one special marine-derived fungus, *Acremonium chrysogenum* has made irreplaceable contributions to controlling the bacterial infections and saving countless patients for production of the *β*-lactam antibiotic cephalosporin C (CPC) and its derivatives [5]. The genomic sequences and annotation of *A. chrysogenum* was first completed in 2014, and a total of 42 secondary metabolite biosynthetic gene clusters, including 14 polyketide synthetase (PKS) clusters, 10 terpene synthase clusters, 8 hybrid clusters, 7 nonribosomal peptide synthetase clusters and 3 non-identified secondary metabolite clusters, were predicted [6].

Sorbicillinoids are a class of structurally diverse hexaketide metabolites with a characteristic sorbyl side chain residue [7,8,9]. They were first isolated from *Penicillium notatum* in 1948 and structurally elucidated by Cram and Tishler [10,11]. Up until now, more than 159 naturally occurring sorbicillinoids have been isolated and have displayed good biological activities in cytotoxic, antimicrobial and phytotoxic activities [7,8,9]. Because free radicals play an important role in the development of aging and many diseases, including cancer, arthritis and atherosclerosis, exploring the novel radical scavengers is crucial for developing new drugs to slow down the aging process and treat these diseases. Some sorbicillinoid derivatives have shown great antioxidative application prospects, such as bisorbicillinol (ED_50_ = 31.4 μM) and bisorbibetanone (ED_50_ = 62.5 μM), etc. [8]. Additionally, there is an urgent need to find more novel compounds for the emergence of microbial resistance. Some sorbicillinoids showed significant antimicrobial activity, indicating their potential as candidates [7]. Meanwhile, the sorbicillinoid biosynthetic gene clusters from *Penicillium* *chrysogenum* and *Trichoderma reesei* have been identified and their biosynthetic pathway has been partially revealed [12,13,14]. Generally, two PKSs SorA and SorB are responsible for the formation of sorbicillin and dihydrosorbicillin, which are then oxidative dearomatized to give sorbicillinol and dihydrosorbicillinol by the FAD-dependent monooxygenase SorC, respectively [15]. Sorbicillinol is regarded as the precursor of most sorbicillinoids since it is condensed with its derivatives or other compounds to form the dimeric and hybrid sorbicillinoids by Diels-Alder or Michael-addition-like reactions [16,17]. The sorbicillinoid biosynthetic gene cluster in *A. chrysogenum* has been regarded as the most ancient, based on evolutionary origin, and carries more modifier than other species [13], and disruption of these two PKS encoding genes results in the abolishment of sorbicillinoids [18]. However, there is lack of a systematic investigation about sorbicillinoids produced by *A. chrysogenum*.

**Figure 1:**
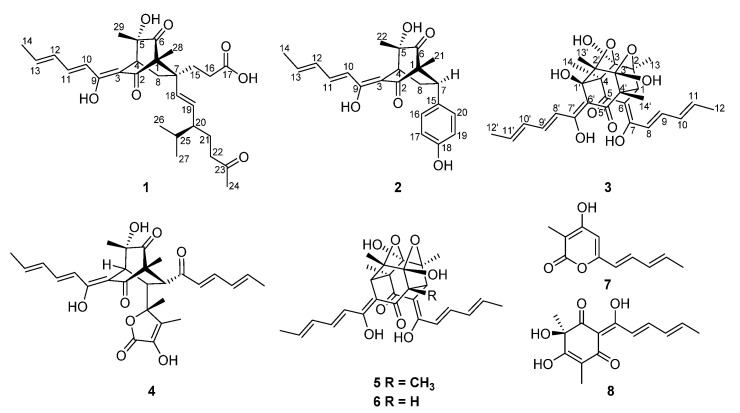
Structures of compounds **1**–**8**.

Based on the chemical investigations in this study, the resulting crude extracts of *A. chrysogenum* C10 from the rice solid fermentation, which has a higher accumulation of compounds and reproducibility than submerged fermentation [19], had afforded three structurally unique compounds: acresorbicillinols A–C (**1**–**3**) and five known sorbicillinoids including trichotetronine (**4**) [20], trichodimerol (**5**) [21], demethyltrichodimerol (**6**) [21], trichopyrone (**7**) [22] and oxosorbicillinol (**8**) [20] (Figure 1). Compounds **1**–**8** were evaluated for their DPPH radical scavenging abilities and antimicrobial activities. In addition, the boundary of sorbicillinoid biosynthetic gene cluster (*Acsor*) was confirmed and its biosynthetic pathway was proposed. This study reported the isolation, structural elucidation and bioactivities of the isolated compounds from *A. chrysogenum* C10.

## 2. Materials and Methods

### 2.1. General Experimental Procedure

Optical rotations, ECD spectra, UV and IR data were measured on the Austria Anton Paar MCP 200 Automatic Polarimeter, the Applied Photophysics Chirascan circular dichroism spectrometer, the Thermo Scientific GENESYS 10S UV-Vis and the Thermo Scientific Nicolet IS5 spectrophotometers, respectively. HRESIMS data and MS were obtained using an Agilent 6520B Q-TOF Mass instrument equipped with an ESI source. All MS experiments were performed in positive ion mode. NMR data were acquired with the AVANCE-500 spectrometer (Bruker, Bremen, Germany) using solvent signals (CD_3_OD, *δ*_H_ 3.30/*δ*_C_ 49.9, DMSO, *δ*_H_ 2.50, 3.30/*δ*_C_ 39.5, and CDCl_3_, *δ*_H_ 7.26/*δ*_C_ 77.16) as references. Octadecylsilyl (ODS, 50 μm, YMC Co., Ltd. Japan) and Sephadex^TM^ LH-20 (Cytiva, Uppsala, Sweden) were used for column chromatography. High performance liquid chromatography (HPLC) was performed on the SHIMADZU LC20AT system equipped with UV diode array detector using the Thermo Hypersil Gold-C18 columns (5 μm, 250 mm × 4.6 mm) at a flow rate of 1 mL/min. For semi-preparative HPLC, Waters 1525 system equipped with the UV/Visible detector and the Thermo Hypersil Gold-C18 columns (5 μm, 250 mm × 10 mm) was used and performed at a flow rate of 2 mL/min. Solvents including methanol and ethyl acetate (EtOAc) for extraction and chromatographic separation were analytical grade. HPLC-grade solvents (acetonitrile and formic acid) were used for the HPLC and semi-preparative HPLC analysis.

### 2.2. Fungal Materials and Fermentation

One high CPC-producing strain of *A. chrysogenum* C10 (ATCC 48272) was released by PanLab. This fungus was inoculated on the rice solid medium in 500 mL Erlenmeyer flasks containing 80 g of rice and 120 mL of H_2_O, and cultivated at 28 °C for 7 days for the production of sorbicillinoids. A total of 10 kg fermentation sample was harvested.

### 2.3. Extraction and Isolation

The rice solid fermentation of *A. chrysogenum* was extracted with EtOAc (3 × 5 L) under the ultrasonication processing. The organic solvents were filtered and evaporated by the vaccum to get the crude extracts (25 g). Extracts were fractionated by ODS reverse silica gel using the gradient MeOH/H_2_O (*v*/*v*, 10%, 20%, 30%, 40%, 50%, 55%, 60%, 65%, 70%, 75%, 80%, 85%, 90%, 95%, 100%) to afford 15 fractions (Fr.1–Fr.15). Fr.8 (MeOH/H_2_O (*v*/*v*, 65%)) (150 mg) was further subjected to the Sephadex^TM^ LH-20 and eluted with MeOH to give 30 subfractions. Fr.8–24 (30 mg) was purified by semi-preparative RP-HPLC using 50% acetonitrile in acidic water (0.1% formic acid) to give compounds **2** (5.0 mg, *t*_R_ = 23 min), **4** (4.0 mg, *t*_R_ = 24.1 min) and **8** (2.0 mg, *t*_R_ = 22.2 min). Fr.10 (MeOH/H_2_O (*v*/*v*, 75%)) (133 mg) was subjected to the Sephadex^TM^ LH-20 and eluted with MeOH to give 30 subfractions. Fr.10–12 (56 mg) was purified by semi-preparative RP-HPLC using 58% acetonitrile in acidic water (0.1% formic acid) to yield compounds **1** (3.5 mg, *t*_R_ = 27.2 min) and **6** (2.5 mg, *t*_R_ = 26.4 min). Fr.11 (MeOH/H_2_O (*v*/*v*, 80%)) (220 mg) was subjected to the Sephadex^TM^ LH-20 and eluted with MeOH to give 30 subfractions. Fr.11–24 (60 mg) was purified by semi-preparative RP-HPLC using 60% acetonitrile in acidic water (0.1% formic acid) to yield compounds **3** (10.0 mg, *t*_R_ = 28.5 min) and **5** (2.9 mg, *t*_R_ = 28.3 min). Fr.5 (MeOH/H_2_O (*v*/*v*, 50%)) (96 mg) was subjected to the Sephadex^TM^ LH-20 and eluted with MeOH to give 25 subfractions. Fr.5-16 (10 mg) was purified by semi-preparative RP-HPLC using 37% acetonitrile in acidic water (0.1% formic acid) to yield compound **7** (2.0 mg, *t*_R_ = 17.5 min).

Acresorbicillinol A (**1**): pale yellow solid; [*α*${]}_{D}^{25}$+81 (*c* 0.1, MeOH); UV (MeOH) *λ*_max_ (log *ε*) 223 (3.65), 367 (1.72) nm; ECD (*c* 3.0 × 10^–3^ M, MeOH) *λ*_max_ (Δ*ε*) 200 (+4.51), 228 (–8.29), 270 (–12.89), 315 (–34.60), 352 (+24.06) nm; IR (neat) *ν*_max_ 3399, 2956, 2872, 1722, 1601, 1446, 1381, 1258 cm^−1^; ^1^H and ^13^C NMR, Table 1; HRESIMS at *m*/*z* 501.2850 [M + H]^+^ (calcd for C_29_H_41_O_7_, 501.2847).

Acresorbicillinol B (**2**): pale yellow solid; [*α*${]}_{D}^{25}$ +5 (*c* 0.1, MeOH); UV (MeOH) *λ*_max_ (log *ε*) 221 (2.72), 322 (0.68), 351 (0.54) nm; ECD (*c* 3.0 × 10^–3^ M, MeOH) *λ*_max_ (Δ*ε*) 215 (–14.07), 245 (+36.00), 315 (–75.16) nm, 360 (+13.47) nm; IR (neat) *ν*_max_ 3413, 1724, 1624, 1440, 1378, 1243 cm^−1^; ^1^H and ^13^C NMR, Table 1; HRESIMS at *m*/*z* 369.1696 [M + H]^+^ (calcd for C_22_H_25_O_5_, 369.1697).

Acresorbicillinol C (**3**): bright yellow solid; [*α*${]}_{D}^{25}$ −1048 (*c* 0.1, MeOH); UV (MeOH) *λ*_max_ (log *ε*) 207 (1.82), 37 (2.12), 278 (2.55), 375 (3.23) nm; ECD (*c* 3.0 × 10^–3^ M, MeOH) *λ*_max_ (Δ*ε*) 221 (–22.24), 275 (+38.06), 345 (+51.25) nm, 405 (–88.67) nm; IR (neat) *ν*_max_ 3420, 1664, 1606, 1556, 1412, 1347, 1209 cm^−1^; ^1^H and ^13^C NMR, Table 2; HRESIMS at *m*/*z* 513.2116 [M + H]^+^ (calcd for C_28_H_33_O_9_, 513.2119).

### 2.4. ECD Calculations

Conformational analyses were performed using Maestro 10.2 in the OPLS3 molecular mechanics force-field within an energy window of 5.0 or 3.0 kcal/mol. The conformers were then further optimized with the software package Gaussian 09 at the B3LYP/6-31G(d) level for compounds **1**–**3**, respectively, and the harmonic vibrational frequencies were also calculated to confirm their stability. The TDDFT methods at the CAM-B3LYP/6-31G(d) and B3LYP/6-31G(d) level were applied to calculate the 60 lowest electronic transitions to obtain conformers in a vacuum, respectively. The Gaussian function was applied to simulate the ECD spectrum of the conformers. The calculated ECD spectra were obtained according to the Boltzmann weighting of each conformer’s ECD spectrum [23].

### 2.5. Antimicrobial Activity Assay

The bacterial strains (*Staphylococcus aureus* CGMCC 1.89, *Pseudomonas aeruginosa* ATCC 15692) and the fungal strains (*Cryptococcus neoformans* W1585, *Candida albicans* SC5314) were used in this study. The concentration of 50 mM compounds was prepared using dimethyl sulfoxide (DMSO). The bacterial and fungal strains were streaked onto Mueller–Hinton Agar (MHA) and Potato Dextrose Agar (PDA) for growth at 37 °C and 28 °C, respectively. Single colony was picked and adjusted to 2 × 10^5^ CFU/mL by Mueller–Hinton Broth (MHB) or Potato Dextrose Broth (PDB). The stock solutions of compounds were diluted into 500, 250, 125, 62.5 and 31.25 μM by MHB or PDB, successively. Fifty microliters of serial dilutions of each compound and 50 μL of microbial suspension were added to the 96-well plates and incubated at 37 °C or 28 °C for 24 h until the results were recorded. IC_50_ was defined as the half maximal inhibitory concentrations of the compounds that inhibited the visible microbial growth after 24 h of incubation. Ampicillin and amphotericin B were used as the positive control for detecting the activities of these compounds against bacteria and fungi, respectively.

### 2.6. DPPH Radical Scavenging Assay

The DPPH radical scavenging activity of the compounds was carried out as previously described [24,25]. The modified parameter was the reaction time from 0.5 h to multiple time-points including 0.5, 1, 4, 6, 8 and 24 h. Ascorbic acid and ethanol were used as the positive and negative control, respectively. All experiments were replicated at least three times.

### 2.7. RNA Isolation and Real-Time RT-PCR Analysis

The mycelia of *A. chrysogenum* C10 grown on the modified MDFA medium were collected at different time-points [26]. RNA isolation and real-time RT-PCR were performed as described previously [27,28]. All primers used in this study were listed in Table S1.

## 3. Results and Discussion

### 3.1. Isolation and Structure Elucidation

**Figure 2:**
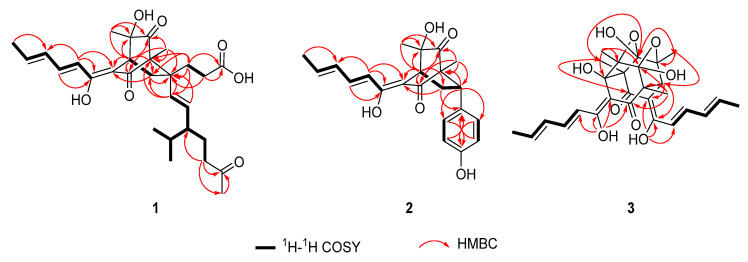
Key COSY and HMBC correlations of compounds **1**–**3**.

Acresorbicillinol A (**1**) was obtained as a pale yellow solid, and its molecular formula was established as C_29_H_40_O_7_ based on HRESIMS data at 501.2850 [M + H]^+^ (calcd for C_29_H_41_O_7_, 501.2847), indicating 10 degrees of unsaturation. The IR spectrum indicated the presence of hydroxy (3399 cm^−1^) and ketone (1722 cm^−1^) groups. The ^1^H NMR data (Table 1 and Figure S1) of **1** showed signals for six methyl signals [*δ*_H_ 2.16 (s, H_3_-24), 1.89 (d, *J* = 7.0 Hz, H_3_-14), 1.16 (s, H_3_-28), 1.12 (s, H_3_-29), 0.86 (d, *J* = 7.0 Hz, H_3_-26), and 0.81 (d, *J* = 7.0 Hz, H_3_-27)], five methylene protons [*δ*_H_ 2.42 (m, H-22a), 2.38 (m, H-8a), 2.30 (m, H-22b), 2.16 (m, H_2_-16), 1.97 (dd, *J* = 13.3, 2.8 Hz, H-8b), 1.81 (td, *J* = 13.2, 4.8 Hz, H-15a), 1.64 (m, H-21a), 1.50 (m, H-15b), and 1.23 (m, H-21b)], three methine protons [*δ*_H_ 3.18 (t, *J* = 2.8 Hz, H-4), 1.68 (m, H-20), and 1.54 (m, H-25)], six olefinic protons [*δ*_H_ 7.26 (dd, *J* = 14.6, 10.9 Hz, H-11), 6.42 (d, *J* = 14.6 Hz, H-10), 6.39 (dd, *J* = 14.6, 10.9 Hz, H-12), 6.20 (dq, *J* = 14.6, 7.0 Hz, H-13), 5.18 (d, *J* = 15.6 Hz, H-18), and 5.13 (dd, *J* = 15.6, 9.0 Hz, H-19)]. Detailed interpretation of the ^13^C NMR and HSQC data (Table 1, Figures S2 and S4) of **1** revealed the presence of 29 carbon resonances corresponding to six methyls, five sp^3^ methylenes, three sp^3^ methines, six sp^2^ methines, three sp^3^ quarternary carbons with one oxygenated, two sp^2^ non-protonated carbons and four carbonyl carbons (*δ*_C_ 212.4, 212.3, 200.3 and 178.3, respectively). These data accounted for all ^1^H and ^13^C NMR resonances of **1** except for three unobserved exchangeable protons, suggesting that **1** was a bicyclic compound. The planar structure of **1** was assigned through detailed analysis of the ^1^H-^1^H COSY and HMBC correlations (Figure 2, Figures S3 and S5). The ^1^H-^1^H COSY (Figure 2) correlations of H-10/H-11/H-12/H-13/H_3_-14, combined with the HMBC correlations from H-10 to the olefinic carbons C-3 (*δ*_C_ 112.3) and C-9 (*δ*_C_ 167.6) and from H-11 to C-9, suggested the presence of the enolic sorbyl side chain. The HMBC correlations (Figure 2) from H-4 to C-3, the sp^3^ quarternary carbon C-5 (*δ*_C_ 75.4) and two ketone carbons C-2 (*δ*_C_ 200.3) and C-6 (*δ*_C_ 212.3), from H_3_-28 to the sp^3^ quarternary carbon C-1 (*δ*_C_ 70.3), C-2 and C-6, and from H_3_-29 to C-4, C-5, and C-6 permitted the completion of the cyclohexandione ring, with the enolic sorbyl unit positioned at C-3 and two methyl groups located at C-1 and C-5, respectively. Meanwhile, the ^1^H-^1^H COSY (Figure 2) correlations of H-18/H-19/H-20/H_2_-21/H_2_-22 and of H-20/H-25/H_3_-26/H_3_-27, as well as the HMBC correlations from H_2_-22 to the ketone carbon C-23 (*δ*_C_ 212.4) and C-24 (*δ*_C_ 30.0), and from H_3_-24 to C-22 and C-23, established the 3-isopropyl-6-oxohept-1-en-1-yl (C-18–C-27) subunit. Moreover, the ^1^H-^1^H COSY (Figure 2) correlations of H_2_-15/H_2_-16, and the HMBC correlations from H_2_-15 and H_2_-16 to the carbonyl carbon C-17 (*δ*_C_ 178.3), indicated that carbonyl carbon C-17 was attached to C-16 directly. Additional HMBC correlations from H_2_-15 to the sp^3^ quarternary carbon C-7 (*δ*_C_ 47.8) and the olefinic carbon C-18 (*δ*_C_ 135.4), from H_2_-16 and H-19 to C-7, and from H-18 to C-7 and C-15, indicated that C-7 was located between C-15 and C-18. Key HMBC correlations from H-4, H_2_-8 and H_3_-28 to C-7, and from H_2_-15 and H-18 to C-1 and C-8, along with the ^1^H-^1^H COSY correlations of H-4/H_2_-8 implied that C-1 and C-8 were all connected to C-7, permitting the completion of the bridged bicyclo [2.2.2]octane-2,6-dione core structure. By consideration of the molecular formula and the chemical shifts of C-5 (*δ*_C_ 75.4) and C-17 (*δ*_C_ 178.3), these two carbons should be hydroxylated. Thus, the planar structure of **1** was established as shown (Figure 1).

**Figure 3:**
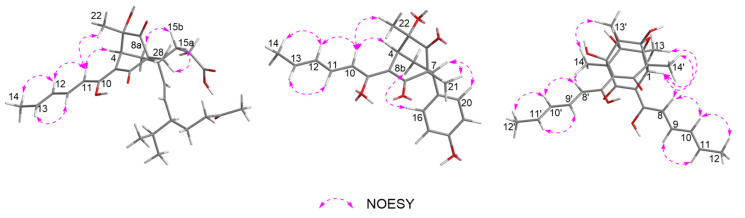
Key NOESY correlations of compounds **1**–**3**.

**Figure 4:**
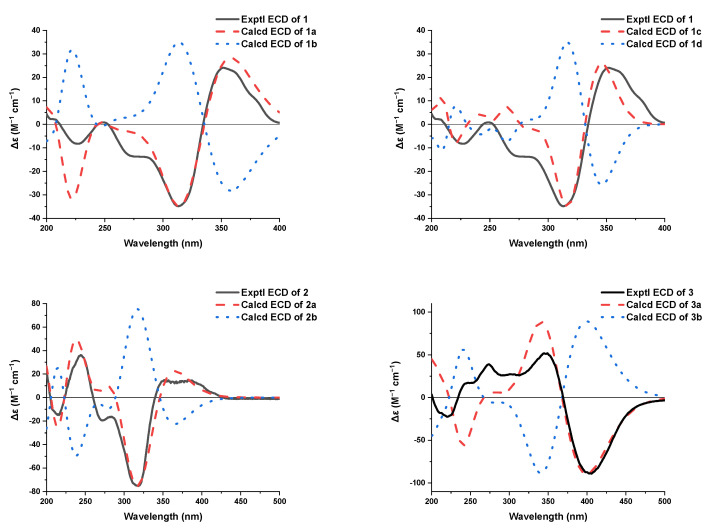
Calculated and experimental ECD spectra of compounds **1**–**3**.

The relative configuration of **1** was determined by NOESY correlations, coupling constants and HMBC correlations. The NOESY correlation (Figure 3 and Figure S6) of H-10 with H-4 assigned the olefin C-3/C-9 as *Z* geometry. The geometry of the conjugated diene was assigned as 10*E*, 12*E* by the large coupling constants (*J*_H-10/H-11_ = 14.6 Hz and *J*_H-12/H-13_ = 14.6 Hz) along with the NOESY correlations of H_3_-14 with H-12 and of H-13 with H-11. The *E* geometry of the C-18/C-19 double bond was also deduced by the large coupling constant between H-18 and H-19 (15.6 Hz). The NOESY correlations of H-10 with H-4 and H_3_-29 suggested that these protons were close in space. Moreover, the strong HMBC correlations from H-8a to C-3 and C-15, and from H-8b to C-5, and the weak correlation from H-8a to C-5, as well as the lack of HMBC correlation from H-8b to C-3 and C-15, indicated that H-8a and C-15 were eclipsed and that H-8b and C-3 were gauche [20,29]. Meanwhile, the NOESY correlations of H_3_-28 with H-15a, and of H-8a with H-15b, assigned the relative configurations of C-1 and C-7. However, the relative configuration for C-20 could not be established by the NOESY data. The absolute configuration for **1** was assigned by a comparison of the experimental and calculated ECD spectra of two pairs of enantiomers, (1*R*,4*S*,5*S*,7*R*,20*S*)-**1** (**1a**), (1*S*,4*R*,5*R*,7*S*,20*R*)-**1** (**1b**), (1*R*,4*S*,5*S*,7*R*,20*R*)-**1** (**1c**), and (1*S*,4*R*,5*R*,7*S*,20*S*)-**1** (**1d**). The ECD calculations were conducted using time-dependent density functional theory (TDDFT) at the CAM-B3LYP/6-31G(d) level. The overall calculated ECD spectrum of **1a**–**1d** was then generated according to Boltzmann weighting of the conformers (Figure S19). For compound **1** the experimental first positive (200 nm), second negative (228 nm), third negative (270 nm), fourth negative (315 nm) and fifth positive (352 nm) Cotton effects compared well with the calculated ECD curve for (1*R*,4*S*,5*S*,7*R*,20*S*)-**1** (**1a**), which showed five corresponding Cotton effects around 200, 222, 270, 315 and 350 nm (Figure 4). Therefore, qualitative analysis of the result allowed the assignment of the absolute configuration of **1** as 1*R*,4*S*,5*S*,7*R*,20*S*.

Acresorbicillinol B (**2**) was obtained as a pale yellow solid. The molecular formula of **2** was assigned as C_22_H_24_O_5_ (11 degrees of unsaturation) based on its HRESIMS data at *m/z* 369.1696 [M + H]^+^ (calcd for C_22_H_25_O_5_, 369.1697). The ^1^H and ^13^C NMR spectroscopic data (Table 1, Figures S7 and S8), in association with the HSQC spectrum (Figure S10), indicated 22 carbon resonances including 3 methyl groups, 1 sp^3^ methylenes, 2 sp^3^ methines, 2 sp^3^ non-protonated carbons with 1 oxygenated, 12 olefinic or aromatic carbons (8 protonated), and 2 carbonyl carbons (*δ*_C_ 211.4, and 199.7, respectively), which were similar to those of **1**. Analysis of the ^1^H-^1^H COSY and HMBC data (Figure 2, Figures S9 and S11) of **2** determined the same bicyclo [2.2.2]octane-2,6-dione moiety with the enolic sorbyl substituted at C-3. However, the substitutes at C-7 of **2** were different from those of **1**. The HMBC correlations from H_2_-8 to C-15 (*δ*_C_ 133.9), from H-7 to C-15, C-16 (*δ*_C_ 130.5) and C-20 (*δ*_C_ 130.5), from H-16/H-20 to C-7 (*δ*_C_ 47.5) and C-18 (*δ*_C_ 157.7) and from H-17/H-19 to C-15 completed the *para*-hydroxyphenyl group located at C-7. On the basis of these data, the planar structure of **2** was established as shown (Figure 1).

The relative stereochemistry of **2** was determined by NOESY correlations and coupling constants as well as by comparison with those of **1** and the known compound sorbicatechol C [30]. The large coupling constants (*J*_H-10/H-11_ = 14.6 Hz and *J*_H-12/H-13_ = 14.6 Hz), along with NOESY correlations (Figure 3 and Figure S12) of H_3_-14 with H-12 and of H-13 with H-11 indicated that the geometry of the conjugated diene was 10*E*, 12*E*. Furthermore, the NOESY correlation (Figure 3) of H-4 with H-10 implied a *Z* geometry of the C3/C9 double bond. Other NOESY correlations of H-10 with H-4 and H_3_-22, and of H-4 with H_3_-22, placed these protons on the same side. While NOESY correlations of H-8b (*δ*_H_ 1.80, ddd, *J* = 13.6, 6.1, 2.7 Hz) with H-16 (H-20), and of H-7 with H_3_-21, combined with the strong HMBC correlations from H-8b to C-5 and C-15, the weak correlation from H-8b to C-3 and lack of HMBC correlation from H-8a to C-5 and C-15 determined the relative stereochemistry of C-7 and C-1 as shown. The absolute configuration of **2** was also determined by a comparison of the experimental and calculated ECD spectra for enantiomers (1*R*,4*S*,5*S*,7*R*)-**2** (**2a**) and (1*S*,4*R*,5*R*,7*S*)-**2** (**2b**). As shown in Figure 4, the experimental ECD spectrum of **2** showed good agreement with the calculated ECD spectrum of (1*R*,4*S*,5*S*,7*R*)-**2** (**2a**), suggesting the absolute configuration of 1*R*,4*S*,5*S*,7*R* for **2**. Thus, the structure of **2** was defined as shown.

Acresorbicillinol C (**3**) was obtained as a bright yellow solid, and its molecular formula was deduced to be C_28_H_32_O_9_ (13 degrees of unsaturation) on the basis of the HRESIMS data at *m*/*z* 513.2116 [M + H]^+^ (calcd for C_28_H_33_O_9_, 513.2119). The IR absorptions suggested the presence of hydroxy (3420 cm^−1^) and ketone (1664 cm^−1^) groups. Its ^1^H NMR data (Table 2 and Figure S13) revealed signals of eight olefinic protons [*δ*_H_ 6.10–7.49], one methine proton [*δ*_H_ 3.71 (s, H-1)] and six methyls [*δ*_H_ 1.89 (d, *J* = 6.8 Hz, H_3_-12’), 1.83 (d, *J* = 6.8 Hz, H_3_-12), 1.31 (s, H_3_-14’), 1.30 (s, H_3_-13), 1.29 (s, H_3_-14), 1.17 (s, H_3_-13’)]. The ^13^C NMR spectrum (Table 2 and Figure S14) and the HSQC data (Figure S16) displayed a total of 28 carbon resonances, which were assignable to 6 methyl groups, 8 sp^2^ methines, 1 sp^3^ methines, 13 non-protonated carbons containing 2 carbonyls (*δ*_C_ 199.3, and 190.9), 4 sp^2^ non-protonated with two oxygenated, 7 sp^3^ non-protonated carbon with 5 oxygenated. These signals (Table 2 and Figures S13−S16) were very similar to those of trichodimerol (**5**) [31,32], except that the proton at the C-1’ position in **5** was changed to a hydroxy moiety in **3**. This was evidenced by the HRESIMS data and HMBC correlations (Figure 2 and Figure S17) from H_3_-13’ and H_3_-14 to C-1’ (*δ*_C_ 78.3). Therefore, **3** was 1’-hydroxylated analogue of **5**.

The relative configuration of **3** was confirmed by NOESY correlations and coupling constants. The NOESY correlations (Figure 3 and Figure S18) of H-9/H-11, of H-8/H-10/H-12, of H-9’/H-11’ and of H-8’/H-10’/H-12’, along with the large coupling constants (*J*_H-8/H-9_ = *J*_H-10/H-11_ = *J*_H-8__’__/H-9__’_ = *J*_H-10__’__/H-11__’_ = 14.6 Hz) suggested the 8*E*, 10*E*, 8’*E* and 10’*E* configurations of the conjugated dienes in the sorbyl side chains. Meanwhile, the NOESY correlations of H-1/H-8 and H_3_-14/H-8’ suggested the *Z* geometry of C-6/C-7 and C-6’/C-7’ double bonds. Furthermore, the NOESY correlations of H-1/H_3_-13, of H_3_-13’/H_3_-14 and of H_3_-14’/H-1 inferred that these protons were in close proximity to their related functional groups, respectively. The similar Cotton effects in the ECD spectra of **3** and **5** deduced the absolute configuration of **3** to be the same as that of **5**, which was further verified by ECD calculations (Figure 4). The calculated ECD curve of (1*S*,2*S*,3*R*,4*R*,1’*R*,2’*S*,3’*R*,4’*R*)-**3** (**3a**) matched well with the experimental data, suggesting the absolute configuration to be 1*S*,2*S*,3*R*,4*R*,1’*R*,2’*S*,3’*R*,4’*R*. Thus, the structure of **3** was defined as depicted.

Except for the new compounds **1**–**3**, the structure of five known sorbicillinoids isolated in this study were confirmed by comparison of the spectroscopic data with those in the literature [20,21,22]. The resulting EtOAc extracts of *A. chrysogenum* cultivated on the rice were screened by HPLC analysis (Figure S20).

### 3.2. Biological Activities Evaluation

To explore the bioactivities of compounds **1**–**8**, their abilities of anti-microorganisms and DPPH radical scavenging were evaluated. The results showed that compounds **2** and **3** exhibited the moderate activities against *S. aureus* and *C. neoformans* with the IC_50_ values of 86.93 ± 1.72 and 69.06 ± 10.50 μM, respectively. However, other compounds did not give IC_50_ value at a concentration below 100 μM (Table 3). No candidate compounds could significantly inhibit the growth of *C. albicans* and* P. aeruginosa*. Compound **3** might function as the *β*-1,6-glucan inhibitor to inhibit the fungal growth as its structural analogue bisvertinolone [33]. Bisvertinolone also exhibited significant inhibitory activity against *S. aureus* with the minimal inhibitory concentration (MIC) value of 30 μg/mL [34]. However, only several monomeric sorbicillinoids from *Scytalidium album* exhibited the weak activity against *C. neoformans* with the MIC value of over 38 μg/mL [35].

**Figure 5:**
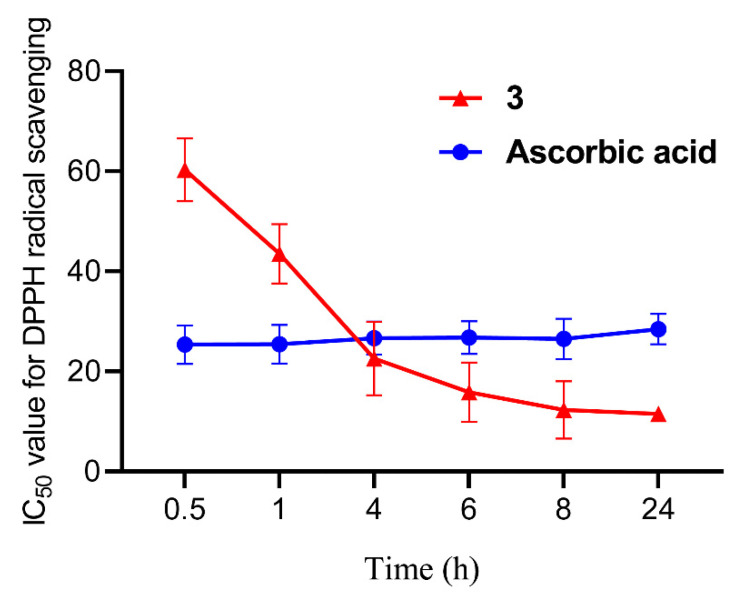
DPPH radical scavenging activity of compound **3** and ascorbic acid as the positive control at 0.5, 1, 4, 6, 8 and 24 h.

Through the DPPH radical scavenging assay, compound **3** exhibited strong activity with the IC_50_ value of 60.29 ± 6.28 μM after standing for 0.5 h, and then we continued to record its radical scavenging activity for 24 h (at 1, 4, 6, 8 and 24 h). Compound **3** gave the significant activity with the IC_50_ values of 43.52 ± 5.93, 22.57 ± 7.34, 15.85 ± 5.94, 12.30 ± 5.74 and 11.53 ± 1.53 μM, respectively, indicating that **3** displays the time-dependent manner for DPPH radical scavenging. Compared with the IC_50_ value of ascorbic acid as the positive control, which was 25.36 ± 3.82 to 28.45 ± 3.04 μM, compound **3** represents one novel DPPH radical scavenging agent (Figure 5 and Table 4). Compound **8** exhibited the radical scavenging activity with the IC_50_ values of 155.40 ± 12.42 and 55.36 ± 14.92 μM for 0.5 and 24 h, respectively. Although the IC_50_ values of **4**, **5** and **6** were over 200 μM for 0.5 h, their radical scavenging activity significantly enhanced at 24 h, and the IC_50_ values were 151.87 ± 15.63, 116.83 ± 3.93 and 102.48 ± 5.04 μM, respectively (Table 4). Compounds **4**, **5**, **6** and **8** also displayed the time-dependent manner as compound **3.** The time-dependent manner of sorbicillinoids for radical scavenging was previously reported, including for oxosorbicillinol, trichotetronine, bisorbicillinolide and methylbisorbibutenolide [22,36,37]. There was a different scavenging values of **4** and **8** between this study and the reports in Hirota’s Lab, and the reaction buffer might be the key determination factor. Additionally, the IC_50_ values of compounds **1**, **2** and **7** exceeded 200 μM, even standing for 24 h, indicating that they did not have DPPH radical scavenging ability (Table 4). DPPH radical scavenging activity of other representative sorbicillinoids has been reported, including for bisorbicillinol, bisvertinolone and bisorbibetanone, which showed ED_50_ values of 31.4, 44.3 and 62.5 μM, respectively [21,37]. To date, compound **3** displayed the best DPPH radical scavenging activity for 24 h among all reported sorbicillinoids.

### 3.3. Determination of Acsor Cluster Boundary and Its Proposed Biosynthetic Pathway of Sorbicillinoid

**Figure 6:**
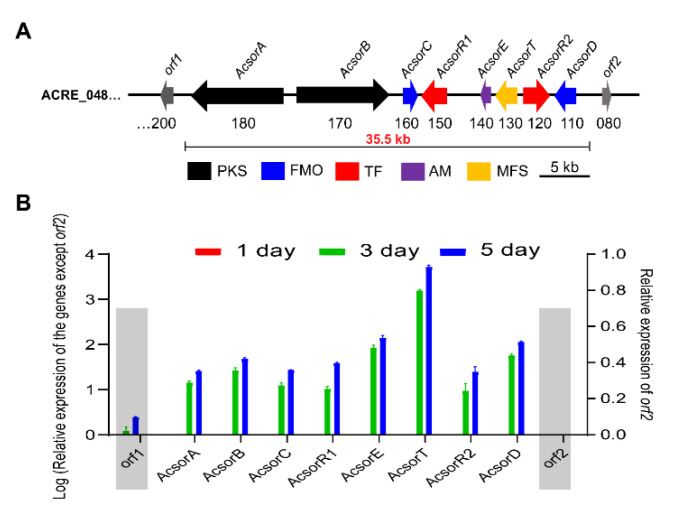
(**A**) Organization of the sorbicillinoid biosynthetic gene cluster. FMO, FAD-dependent monooxygenase; PKS, polyketide synthase; TF, transcriptional factor; AM, auxiliary modifier; MFS, major facilitator superfamily transporter. (**B**) Transcriptional profiles of the *Acsor* genes during fermentation.

To confirm the boundary of the sorbicillinoid biosynthetic gene cluster, the total RNA was isolated from *A. chrysogenum* C10 after incubation in the modified MDFA medium (also producing sorbicillinoids as in the rice solid medium) for 1, 3 and 5 days, and used as a template for real-time RT-PCR, the transcriptions of all 10 genes, including *orf2* (ACRE_048080), *AcsorD* (ACRE_048110), *AcsorR2* (ACRE_048120), *AcsorT* (ACRE_048130), *AcsorE* (ACRE_048140), *AcsorR1* (ACRE_048150), *AcsorC* (ACRE_048160), *AcsorB* (ACRE_048170), *AcsorA* (ACRE_048180) and *orf1* (ACRE_048200), were analysed (Figure 6A). Transcriptional results showed that *AcsorA*, *AcsorB*, *AcsorC*, *AcsorD*, *AcsorE*, *AcsorT*, *AcsorR1* and *AcsorR2* displayed a similar transcriptional pattern. In other words, the transcriptional level gradually increases during the fermentation. However, *orf2* was silent during fermentation. Although *orf1* was transcribed, the transcriptional trend was significantly different from other genes in the *Acsor* cluster. Thus, *orf1* and *orf2* are considered to be situated outside the *Acsor* cluster (Figure 6B). Combining with the results from bioinformatic analysis, a 35.5 kb *Acsor* cluster was identified that contains eight genes encoding one high-reducing polyketide synthase AcsorA, one non-reducing PKS AcsorB, two FAD-dependent monooxygenases AcsorC and AcsorD, one major facilitator superfamily transporter AcsorT, two putative regulators AcsorR1 and AcsorR2 and one putative serine hydrolase AcsorE.

**Figure 7:**
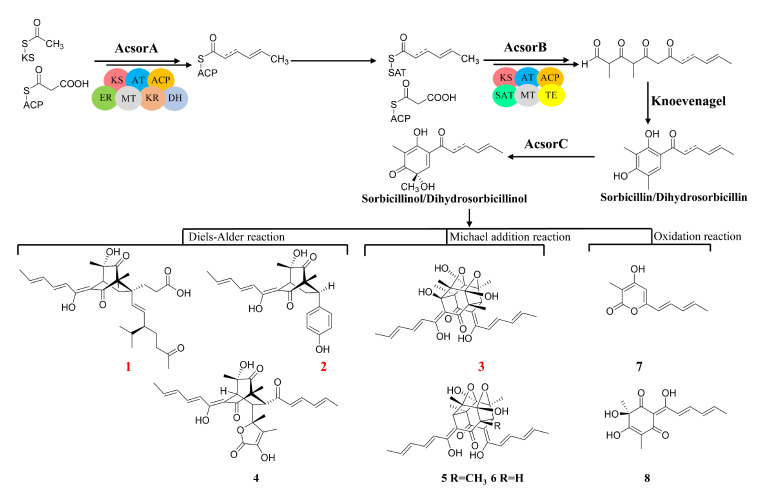
Proposed biosynthetic pathway of compounds **1**–**8**.

Based on the confirmation of *Acsor* cluster, the biosynthetic pathway of compounds **1**–**8** was proposed. Sorbicillinoid biosynthesis starts from the formation of the polyketide backbone via condensation of acetate units catalyzed by AcsorA and AcsorB to generate sorbicillin and dihydrosorbicillin, and then they are oxidative dearomatized by AcsorC to form the common precursor-sorbicillinol and dihydrosorbicillinol. Sorbicillinol and its derivatives can be converted to **1**, **2** and **4** by a Diels–Alder reaction. Compounds **3**, **5** and **6** were biosynthesized by a Michael addition of sorbicillinol. Compounds **7** and **8** could be formed from sorbicillinol by an oxidation reaction (Figure 7). The structure diversification of sorbicillinoid derivatives was likely due to the multi-functions of *AcsorD* in *A. chrysogenum*.

## 4. Conclusions

In summary, eight sorbicillinoid derivatives including three new ones, acresorbicillinols A–C (**1**–**3**), were isolated from the marine-derived fungus *A. chrysogenum*. The absolute configurations of compounds **1**–**3** were determined by ECD calculations. Compound **3** exhibited strong DPPH radical scavenging, indicating that it can be regarded as one novel DPPH radical scavenging agent. Compounds **2** and **3** exhibited the moderate activities against *S. aureus* and *C. neoformans*, respectively. Meanwhile, the boundary of the *Acsor* cluster was confirmed and the biosynthetic pathway of compounds **1**–**8** was also proposed. This study suggests that *A. chrysogenum* is a potential pool for novel sorbicillinoids and radical scavenging agents.

## Figures and Tables

**Table 1:** ^1^H NMR (500 MHz) and ^13^C NMR data (125 MHz) for **1** and **2**.

Position	1 ^a^		2 ^a^	
	*δ*_H_ (*J* in Hz)	*δ*_C_	*δ*_H_ (*J* in Hz)	*δ*_C_
1		70.3, qC		66.7, qC
2		200.3, qC		199.7, qC
3		112.3, qC		113.8, qC
4	3.18, t (2.8)	41.5, CH	3.30, t (2.7)	42.3, CH
5		75.4, qC		75.2, qC
6		212.3, qC		211.4, qC
7		47.8, qC	3.09, dd (10.6, 6.1)	47.5, CH
8a	2.38, m	30.6, CH_2_	3.00, ddd (13.6, 10.6, 2.7)	32.7, CH_2_
8b	1.97, dd (13.3, 2.8)		1.80, ddd (13.6, 6.1, 2.7)	
9		167.6, qC		167.7, qC
10	6.42, d (14.6)	119.5, CH	6.48, d (14.6)	119.6, CH
11	7.26, dd (14.6, 10.9)	142.9, CH	7.37, dd (14.6, 11.0)	143.3, CH
12	6.39, dd (14.6, 10.9)	132.3, CH	6.41, dd (14.6, 11.0)	132.3, CH
13	6.20, dq (14.6, 7.0)	140.0, CH	6.23, dq (14.6, 7.0)	140.1, CH
14	1.89, d (7.0)	18.9, CH_3_	1.90, d (7.0)	18.9, CH_3_
15a 15b	1.81, td (13.2, 4.8) 1.50, m	34.1, CH_2_		133.9, qC
16	2.16, m	31.4, CH_2_	6.80, d (8.4)	130.5, CH
17		178.3, qC	6.67, d (8.4)	116.2, CH
18	5.18, d (15.6)	135.4, CH		157.7, qC
19	5.13, dd (15.6, 9.0)	135.9, CH	6.67, d (8.4)	116.2, CH
20	1.68, m	50.6, CH	6.80, d (8.4)	130.5, CH
21a 21b	1.64, m 1.23, m	27.2, CH_2_	0.80, s	11.4, CH_3_
22a 22b	2.42, m 2.30, m	42.4, CH_2_	1.21, s	24.0, CH_3_
23		212.4, qC		
24	2.16, s	30.0, CH_3_		
25	1.54, m	33.4, CH		
26	0.86, d (7.0)	21.2, CH_3_		
27	0.81, d (7.0)	19.7, CH_3_		
28	1.16, s	7.4, CH_3_		
29	1.12, s	24.5, CH_3_		

^a^ Recorded in CD_3_OD.

**Table 2:** ^1^H NMR (500 MHz) and ^13^C NMR data (125 MHz) for **3**.

Position	3 ^b^	
	*δ*_H_ (*J* in Hz)	*δ*_C_
1	3.71, s	53.9, CH
2		78.2, qC
3		107.8, qC
4		59.2, qC
5		190.9, qC
6		100.6, qC
7		167.9, qC
8	6.49, d (14.6)	120.6, CH
9	7.12, dd (14.6, 10.9)	137.8, CH
10	6.38, overlap	131.1, CH
11	6.10, (14.6, 6.8)	136.2, CH
12	1.83, d (6.8)	18.4, CH_3_
13	1.30, s	25.2, CH_3_
14	1.29, s	18.8, CH_3_
1’		78.3, qC
2’		78.7, qC
3’		103.5, qC
4’		59.2, qC
5’		199.3, qC
6’		108.0, qC
7’		185.2, qC
8’	7.38, d (14.6)	122.4, CH
9’	7.48, dd (14.6, 10.9)	146.5, CH
10’	6.38, overlap	131.1, CH
11’	6.42, overlap	143.4, CH
12’	1.89, d (6.8)	18.3, CH_3_
13’	1.17, s	22.2, CH_3_
14’	1.31, s	18.8, CH_3_
OH-7	16.38, s	
OH-7’	18.02, s	

^b^ Recorded in DMSO:CDCl_3_ = 3:1.

**Table 3:** Anti-microbial inhibitory activities of compounds **1**–**8**.

Compounds	*S. aureus*	*C. neoformans*
	IC_50_ (μM)	
**1**	>100	>100
**2**	86.93 ± 1.72	>100
**3**	>100	69.06 ± 10.50
**4**	>100	>100
**5**	>100	>100
**6**	>100	>100
**7**	>100	>100
**8**	>100	>100
**Ampicillin**	0.016 ± 0.004	–
**Amphotericin B**	–	0.018 ± 0.003

**Table 4:** DPPH radical scavenging activities of compounds **1**–**8**.

Compounds	IC_50_ Value (μM)					
	0.5 h	1 h	4 h	6 h	8 h	24 h
**1**	>200	>200	>200	>200	>200	>200
**2**	>200	>200	>200	>200	>200	>200
**3**	60.29 ± 6.28	43.52 ± 5.93	22.57 ± 7.34	15.85 ± 5.94	12.30 ± 5.74	11.53 ± 1.53
**4**	>200	>200	>200	>200	>200	151.87 ± 15.63
**5**	>200	>200	>200	>200	>200	116.83 ± 3.93
**6**	>200	>200	>200	>200	197.73 ± 27.70	102.48 ± 5.04
**7**	>200	>200	>200	>200	>200	>200
**8**	155.40 ± 12.42	129.87 ± 12.09	88.38 ± 16.29	77.20 ± 15.38	71.00 ± 14.56	55.36 ± 14.92
**Ascorbic acid**	25.36 ± 3.82	25.42 ± 3.85	26.65 ± 3.29	26.77 ± 3.24	26.48 ± 4.03	28.45 ± 3.04

## Data Availability

All data generated or analyzed in this study are available within the manuscript and are available from the corresponding authors upon request.

## Supplementary Materials

The following supporting information can be downloaded at: https://www.mdpi.com/article/10.3390/jof8050530/s1, Figure S1: ^1^H NMR spectrum of acresorbicillinol A (**1**; 500 MHz, CD_3_OD), Figure S2: ^13^C NMR spectrum of acresorbicillinol A (**1**; 125 MHz, CD_3_OD), Figure S3: ^1^H-^1^H COSY spectrum of acresorbicillinol A (**1**, CD_3_OD), Figure S4: HSQC spectrum of acresorbicillinol A (**1**, CD_3_OD), Figure S5: HMBC spectrum of acresorbicillinol A (**1**, CD_3_OD), Figure S6: NOESY spectrum of acresorbicillinol A (**1**, CD_3_OD), Figure S7: ^1^H NMR spectrum of acresorbicillinol B (**2**; 500 MHz, CD_3_OD), Figure S8: ^13^C NMR spectrum of acresorbicillinol B (**2**; 125 MHz, CD_3_OD), Figure S9: ^1^H-^1^H COSY spectrum of acresorbicillinol B (**2**, CD_3_OD), Figure S10: HSQC spectrum of acresorbicillinol B (**2**, CD_3_OD), Figure S11: HMBC spectrum of acresorbicillinol B (**2**, CD_3_OD), Figure S12. NOESY spectrum of acresorbicillinol B (**2**, CD_3_OD) Figure S13: ^1^H NMR spectrum of acresorbicillinol C (**3**; 500 MHz, DMSO:CDCl_3_ = 3:1), Figure S14: ^13^C NMR spectrum of acresorbicillinol C (**3**; 125 MHz, DMSO:CDCl_3_ = 3:1), Figure S15: ^1^H-^1^H COSY spectrum of acresorbicillinol C (**3**, DMSO:CDCl_3_ = 3:1), Figure S16: HSQC spectrum of acresorbicillinol C (**3**, DMSO:CDCl_3_ = 3:1), Figure S17: HMBC spectrum of acresorbicillinol C (**3**, DMSO:CDCl_3_ = 3:1), Figure S18: NOESY spectrum of acresorbicillinol C (**3**, DMSO:CDCl_3_ = 3:1), Figure S19: ECD conformers of acresorbicillinols A–C (**1**–**3**). Figure S20: HPLC profiles of the extracts from the rice solid medium of *A. chrysogenum* after 7 days fermentation. Tale S1: Primers used in this study.
