# Supplementary material for: Sorbicillinoid Derivatives with the Radical Scavenging Activities from the Marine-Derived Fungus Acremonium chrysogenum C10
Source: J Fungi (Basel). 2022 May 20;8(5):530. doi: 10.3390/jof8050530 (PMC9144096; doi:10.3390/jof8050530)
Supplement: Supplementary file 1 [file jof-08-00530-s001.zip › jof-1721082-supplementary.pdf]

Sorbicillinoid Derivatives with the Radical Scavenging Activities from the Marine-Derived Fungus *Acremonium chrysogenum* C10

Chengbao Duan[1,2,†], Shiyuan Wang[1,2,†], Ruiyun Huo[1,2,†], Erwei Li[3], Min Wang[4], Jinwei Ren[1], Yuanyuan Pan[1,*], Ling Liu[1,2*] and Gang Liu[1,2,*]

1 State Key Laboratory of Mycology, Institute of Microbiology, Chinese Academy of Sciences, Beijing 100101, China; 18813111804@163.com (C.D.); wangshiyuan20@mails.ucas.ac.cn (S.W.); huory@im.ac.cn (R.H.); renjw@im.ac.cn (J.R); panyy@im.ac.cn (Y.P.); liul@im.ac.cn (L.L.); liug@im.ac.cn (G.L.)
2 University of Chinese Academy of Sciences, Beijing 100049, China
3 Institutional Center for Shared Technologies and Facilities, Institute of Microbiology, Chinese Academy of Sciences, Beijing 100101, China; liew@im.ac.cn (E.L.)
4 State Key Laboratory of Microbial Resources, Institute of Microbiology, Chinese Academy of Sciences, Beijing 100101, China; hualuozhisheng@126.com (M.W.)

[†]These authors contributed equally to this work.

*Correspondences:
Yuanyuan Pan, E-mail: panyy@im.ac.cn; Tel.: +86-10-64806113
Ling Liu, E-mail: liul@im.ac.cn; Tel.: +86-10-64807043
Gang Liu, E-mail: liug@im.ac.cn; Tel.: +86-10-64806017

No.	Contents	Pages
1.	Figure S1. ^{1}H NMR spectrum of acresorbicillinol A (**1**; 500 MHz, CD_3OD)	3
2.	Figure S2. ^{13}C NMR spectrum of acresorbicillinol A (**1**; 125 MHz, CD_3OD)	4
3.	Figure S3. ^{1}H-^{1}H COSY spectrum of acresorbicillinol A (**1**, CD_3OD)	5
4.	Figure S4. HSQC spectrum of acresorbicillinol A (**1**, CD_3OD)	6
5.	Figure S5. HMBC spectrum of acresorbicillinol A (**1**, CD_3OD)	7
6.	Figure S6. NOESY spectrum of acresorbicillinol A (**1**, CD_3OD)	8
7.	Figure S7. ^{1}H NMR spectrum of acresorbicillinol B (**2**; 500 MHz, CD_3OD)	9
8.	Figure S8. ^{13}C NMR spectrum of acresorbicillinol B (**2**; 125 MHz, CD_3OD)	10
9.	Figure S9. ^{1}H-^{1}H COSY spectrum of acresorbicillinol B (**2**, CD_3OD)	11
10.	Figure S10. HSQC spectrum of acresorbicillinol B (**2**, CD_3OD)	12
11.	Figure S11. HMBC spectrum of acresorbicillinol B (**2**, CD_3OD)	13
12.	Figure S12. NOESY spectrum of acresorbicillinol B (**2**, CD_3OD)	14
13.	Figure S13. ^{1}H NMR spectrum of acresorbicillinol C (**3**; 500 MHz, DMSO:$CDCl_3$ = 3:1)	15
14.	Figure S14. ^{13}C NMR spectrum of acresorbicillinol C (**3**; 125 MHz, DMSO:$CDCl_3$ = 3:1)	16
15.	Figure S15. ^{1}H-^{1}H COSY spectrum of acresorbicillinol C (**3**, DMSO:$CDCl_3$ = 3:1)	17
16.	Figure S16. HSQC spectrum of acresorbicillinol C (**3**, DMSO:$CDCl_3$ = 3:1)	18
17.	Figure S17. HMBC spectrum of acresorbicillinol C (**3**, DMSO:$CDCl_3$ = 3:1)	19
18.	Figure S18. NOESY spectrum of acresorbicillinol C (**3**, DMSO:$CDCl_3$ = 3:1)	20
19.	Figure S19. ECD conformers of acresorbicillinols A–C (**1**–**3**)	21
20.	Figure S20. HPLC profiles of the extracts from the rice solid medium of *A. chrysogenum* after 7 days fermentation	25
21.	Tale S1: Primers used in this study	26

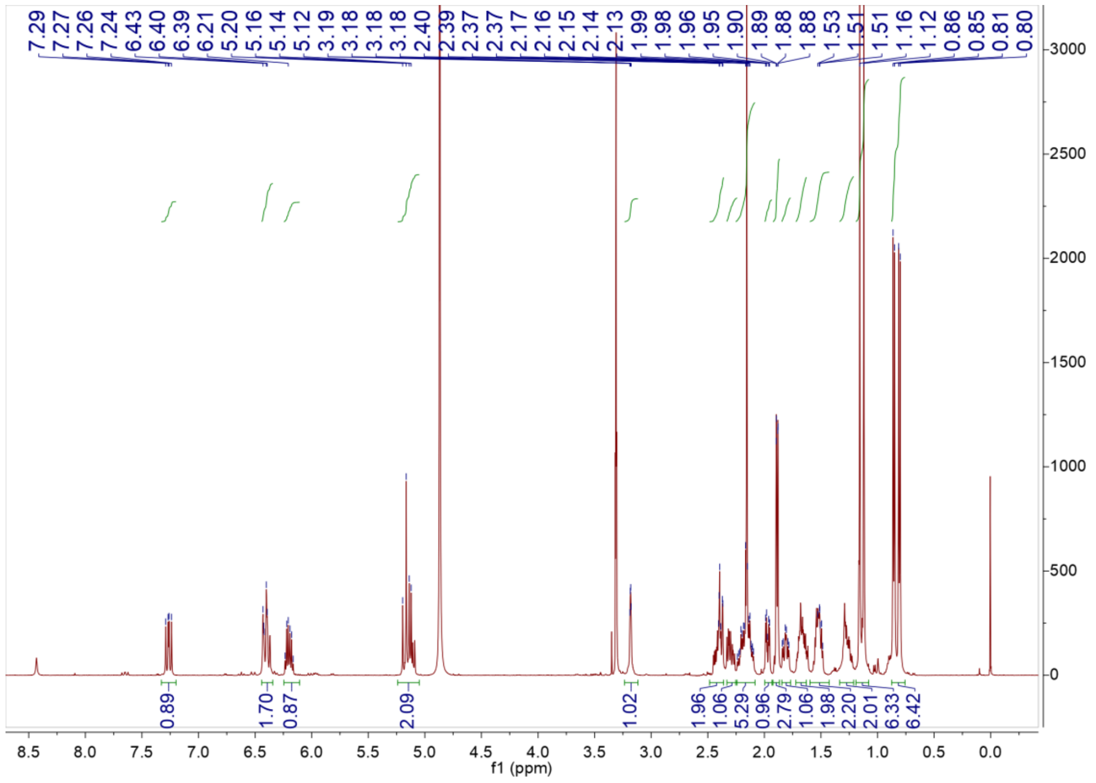

Figure S1. ^{1}H NMR spectrum of acresorbicillinol A (**1**; 500 MHz, CD_3OD)

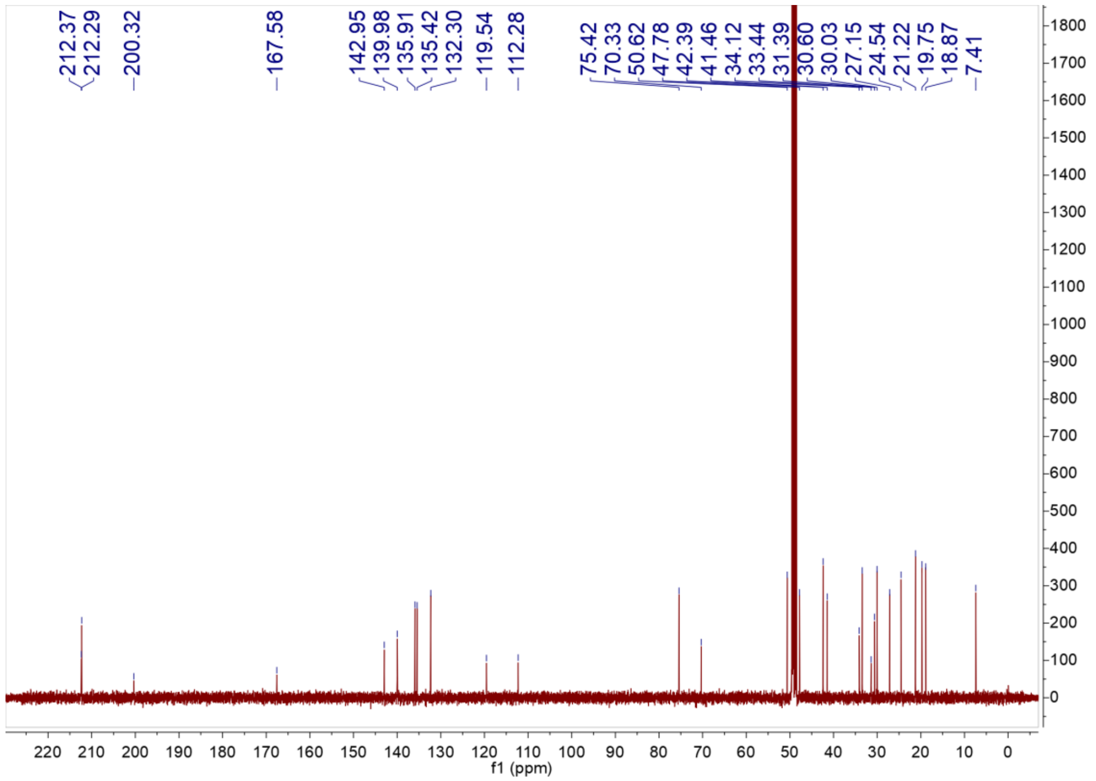

Figure S2. ^{13}C NMR spectrum of acresorbicillinol A (**1**; 125 MHz, CD_3OD)

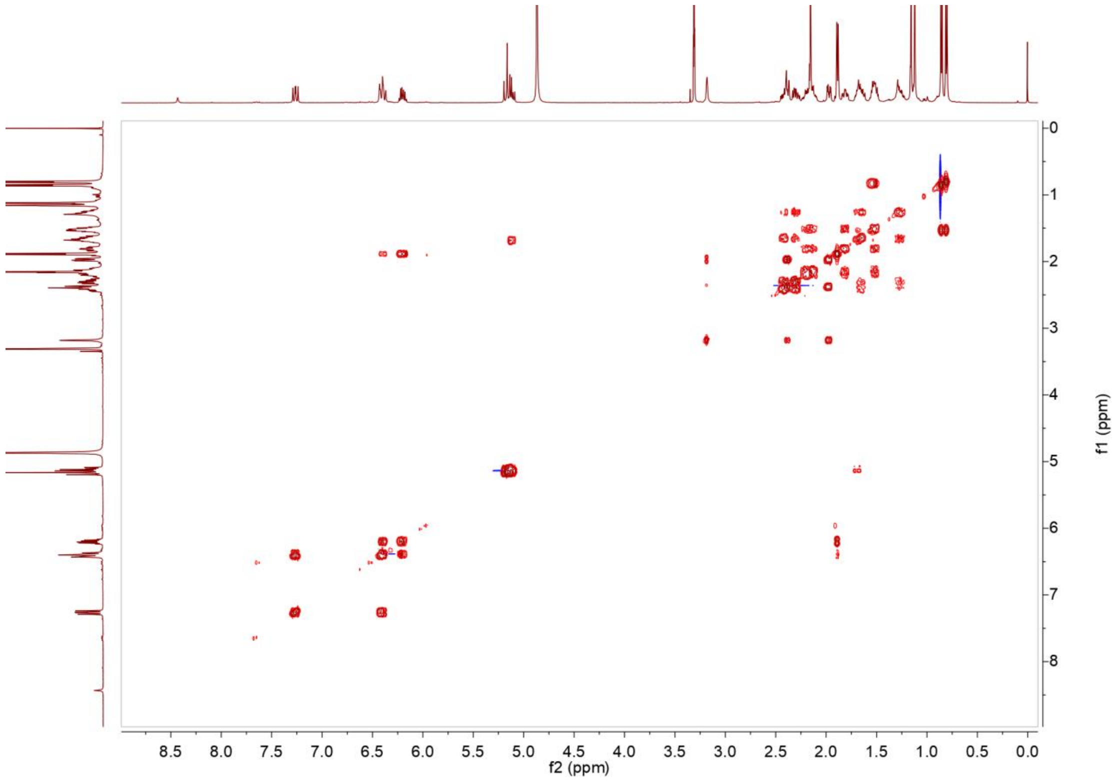

Figure S3. ^{1}H-^{1}H COSY spectrum of acresorbicillinol A (**1**, CD_3OD

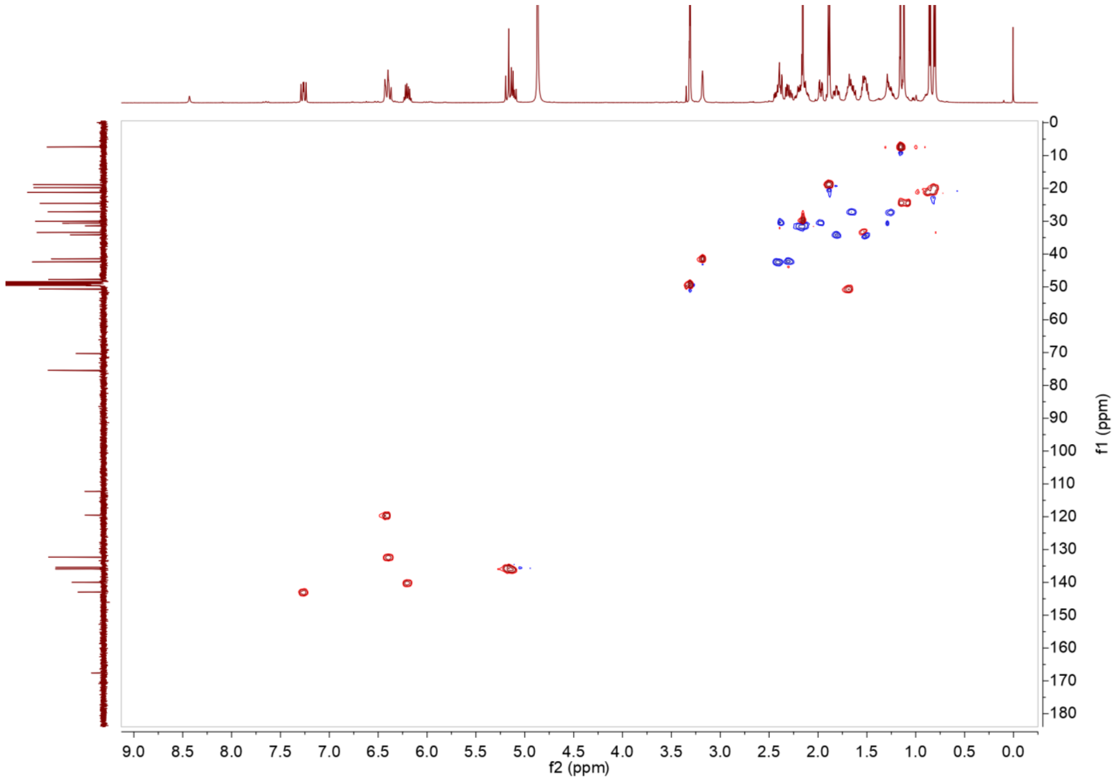

Figure S4. HSQC spectrum of acresorbicillinol A (**1**, CD_3OD)

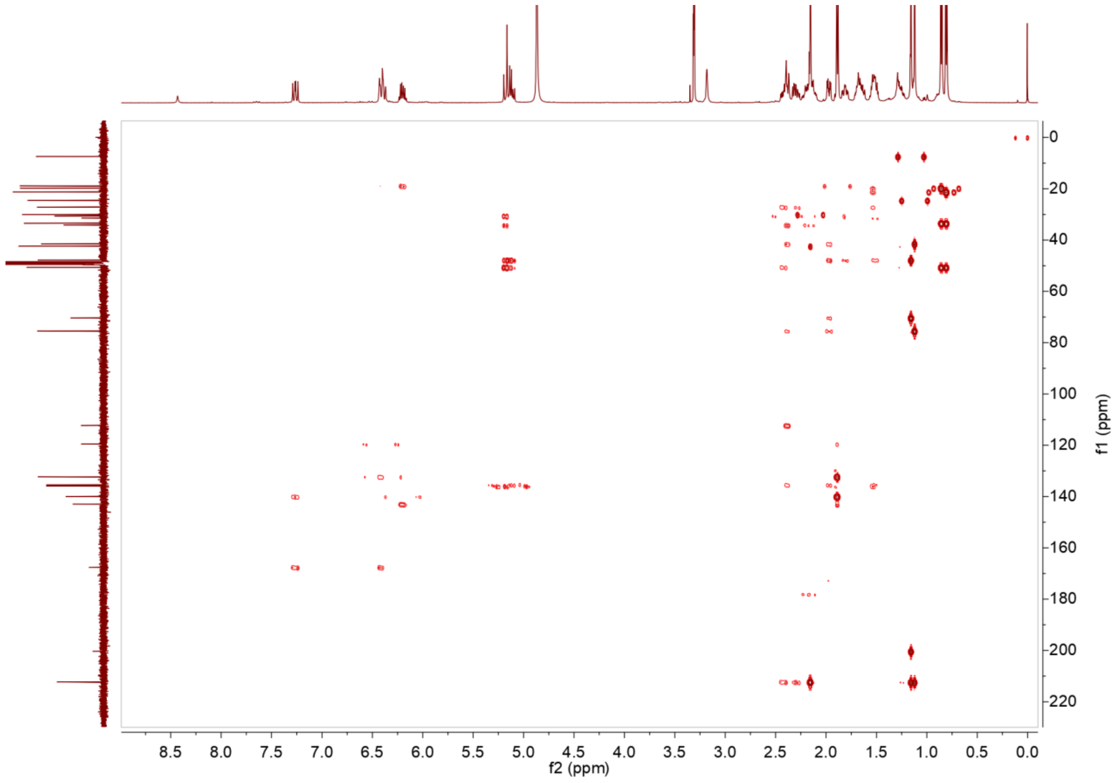

Figure S5. HMBC spectrum of acresorbicillinol A (**1**, CD_3OD)

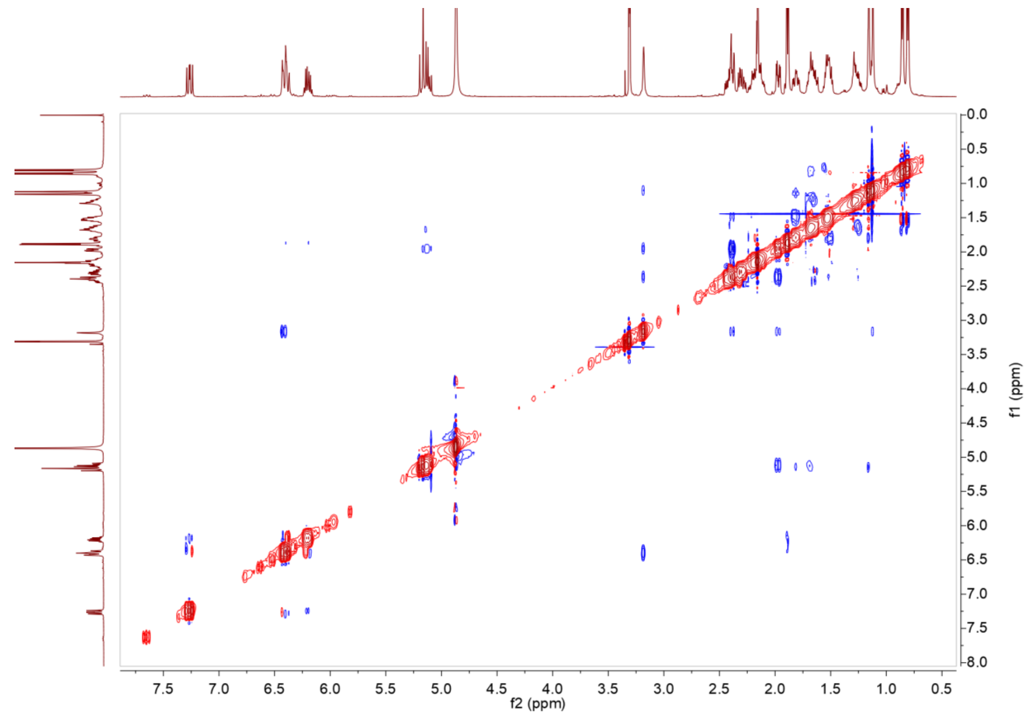

Figure S6. NOESY spectrum of acresorbicillinol A (**1**, CD_3OD)

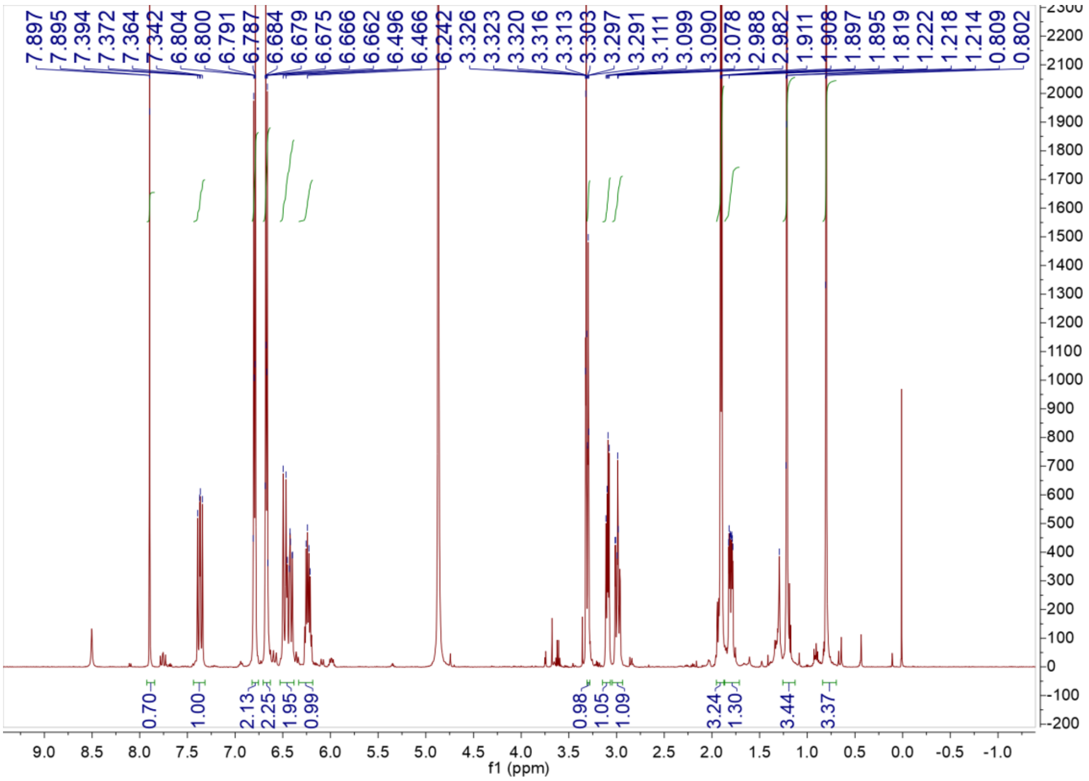

Figure S7. ^{1}H NMR spectrum of acresorbicillinol B (**2**; 500 MHz, CD_3OD)

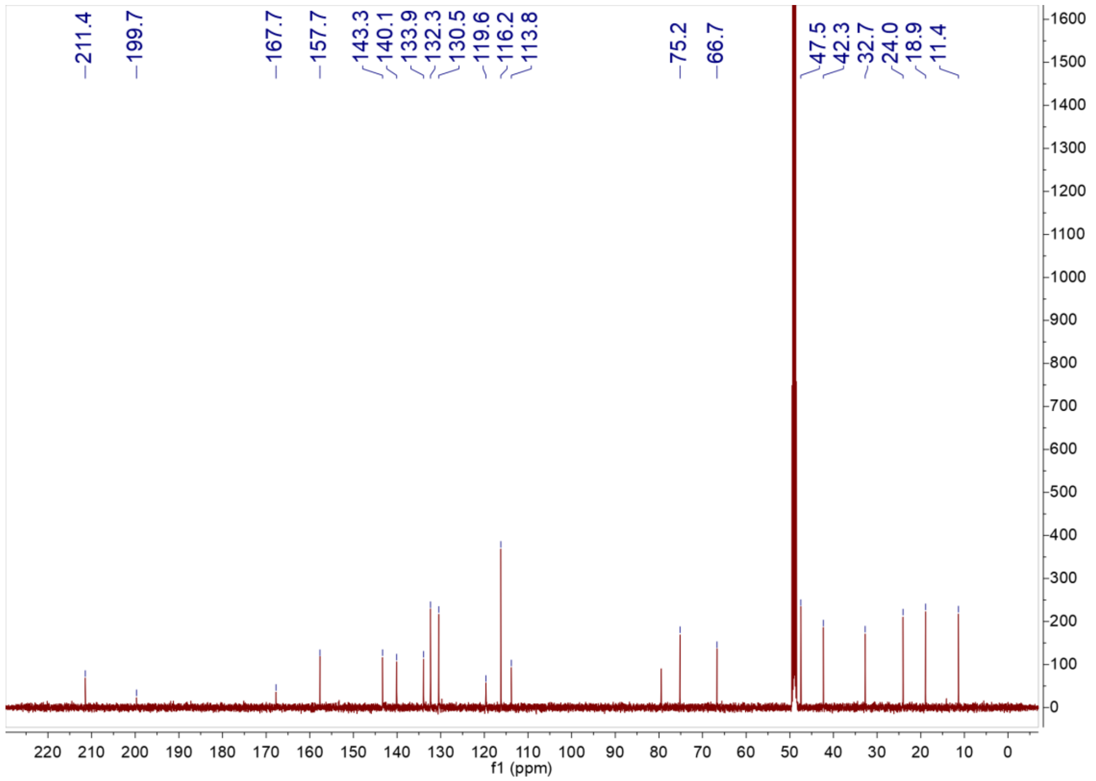

Figure S8. ^{13}C NMR spectrum of acresorbicillinol B (**2**; 125 MHz, CD_3OD)

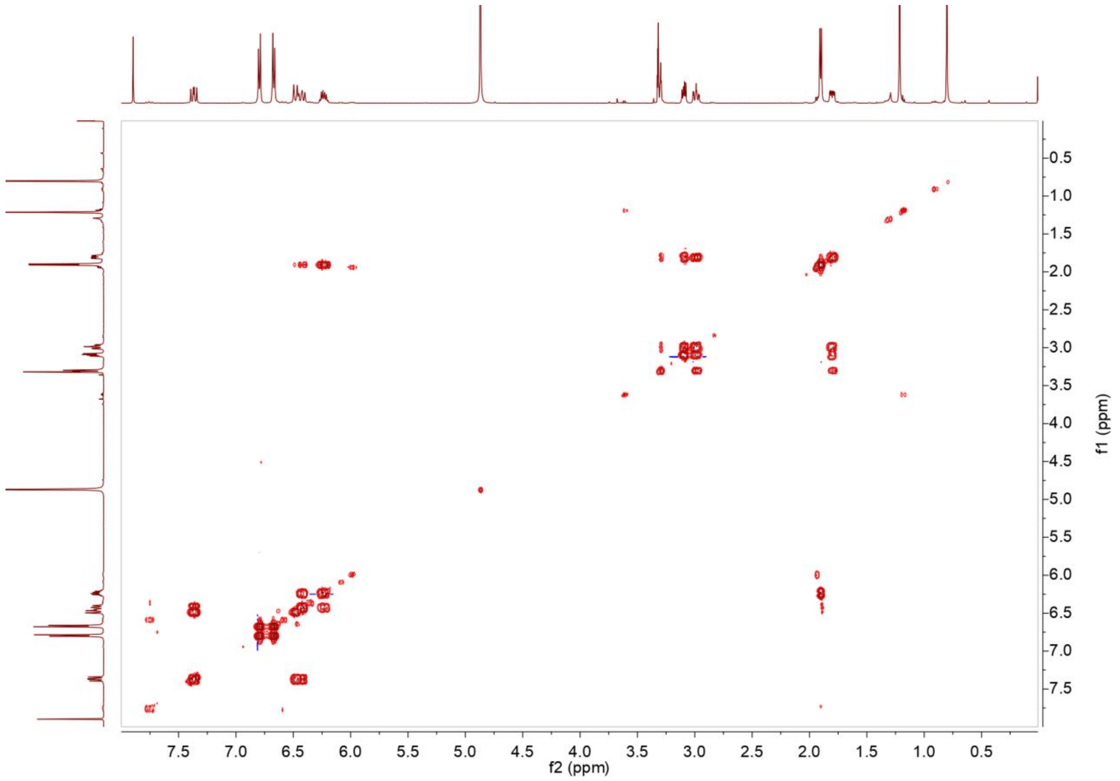

Figure S9. ^{1}H-^{1}H COSY spectrum of acresorbicillinol B (**2**, CD_3OD)

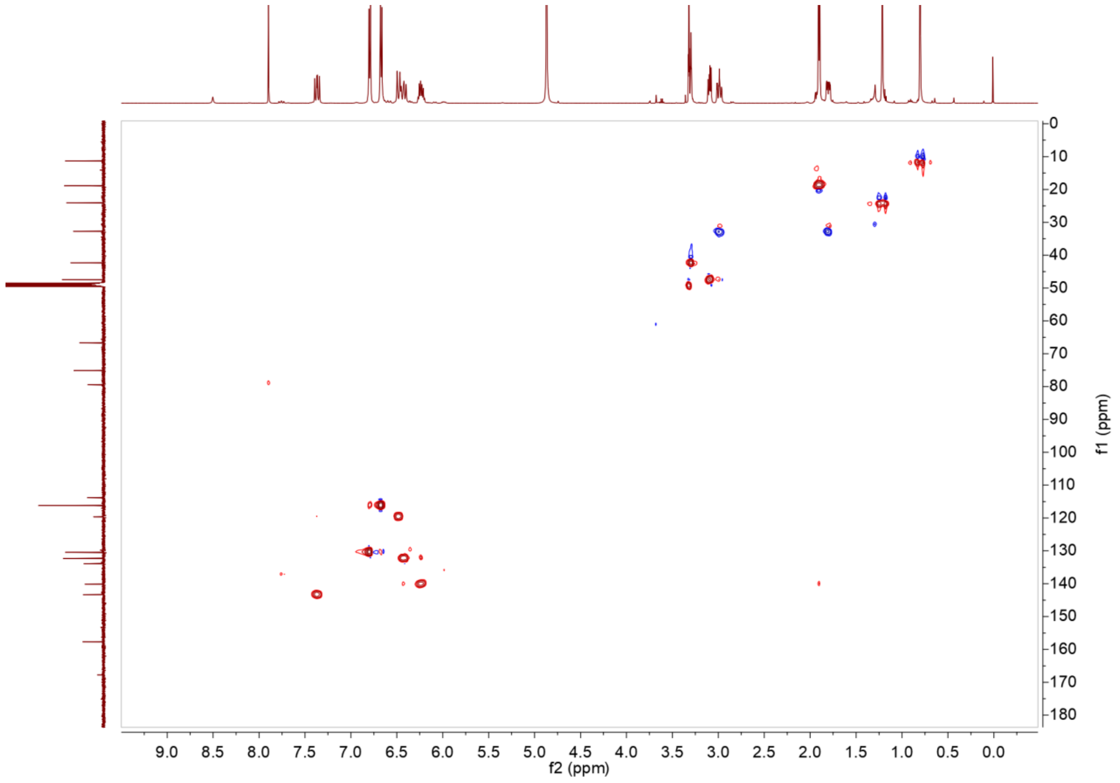

Figure S10. HSQC spectrum of acresorbicillinol B (**2**, CD_3OD)

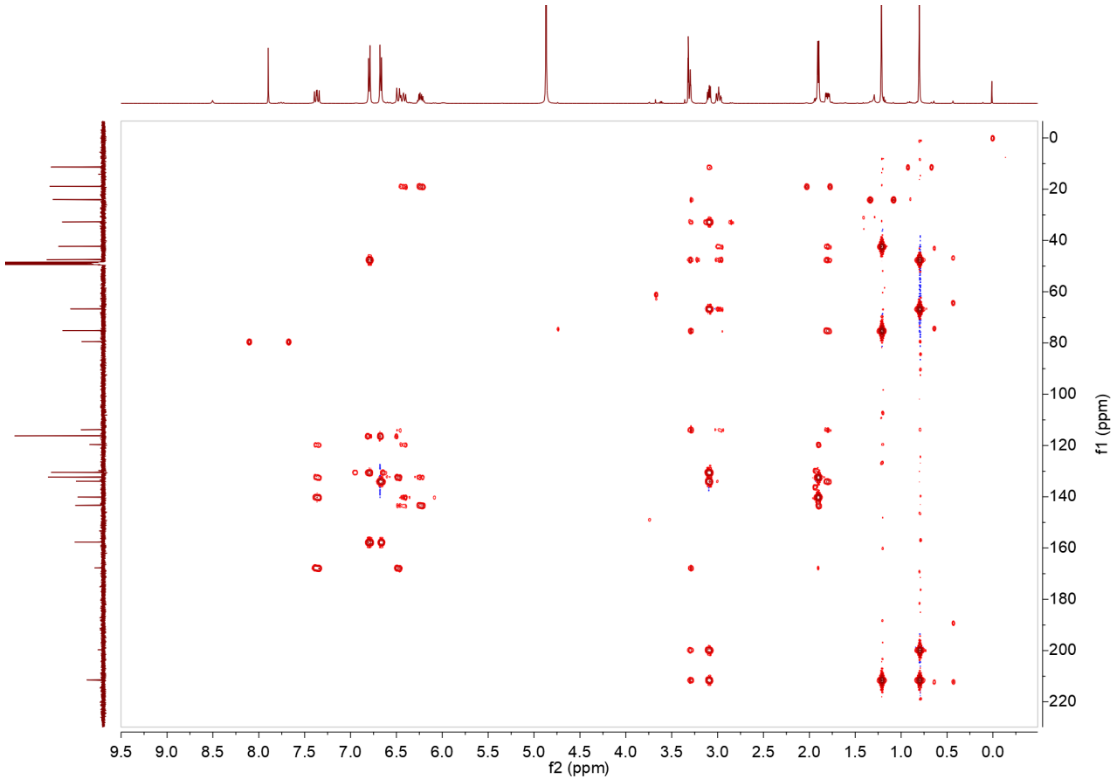

Figure S11. HMBC spectrum of acresorbicillinol B (**2**, CD_3OD)

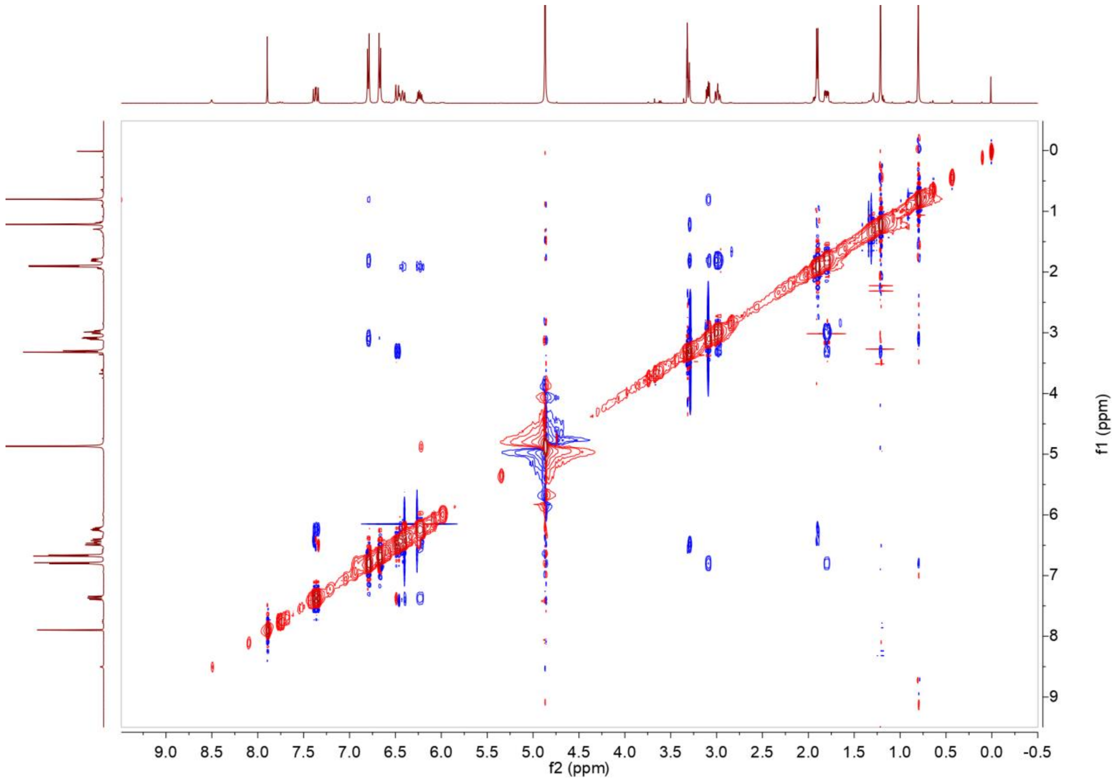

Figure S12. NOESY spectrum of acresorbicillinol B (**2**, CD_3OD)

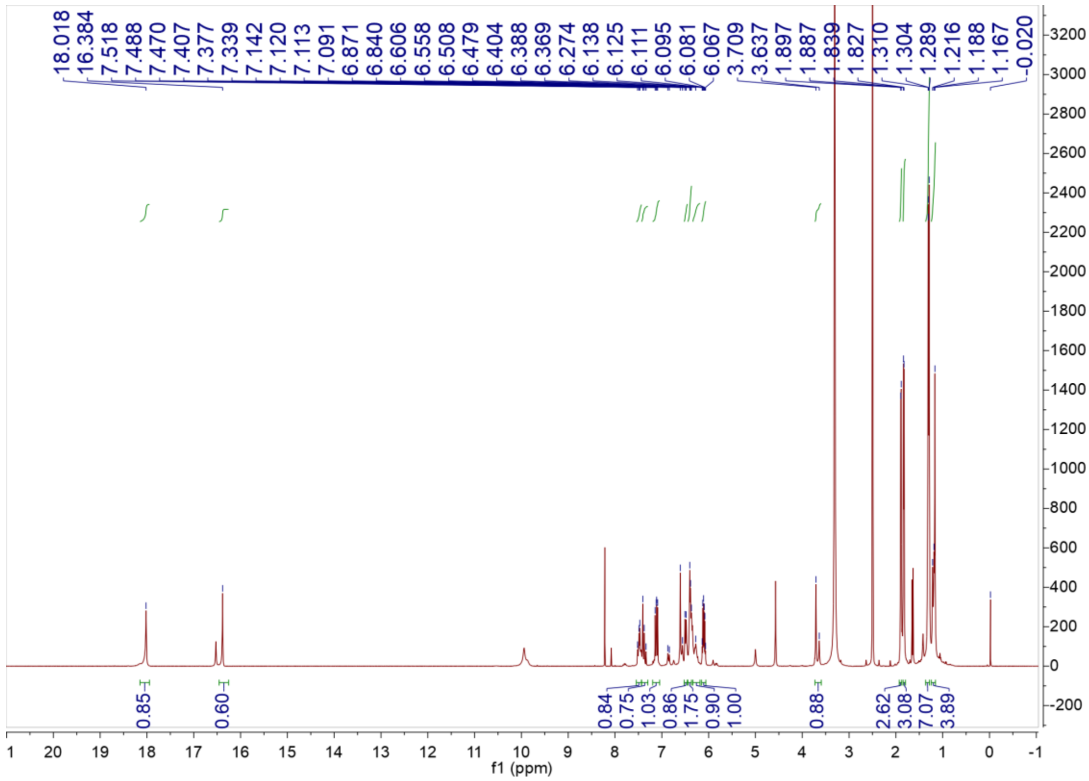

Figure S13. ^{1}H NMR spectrum of acresorbicillinol C (**3**; 500 MHz, DMSO:$CDCl_3$ = 3:1)

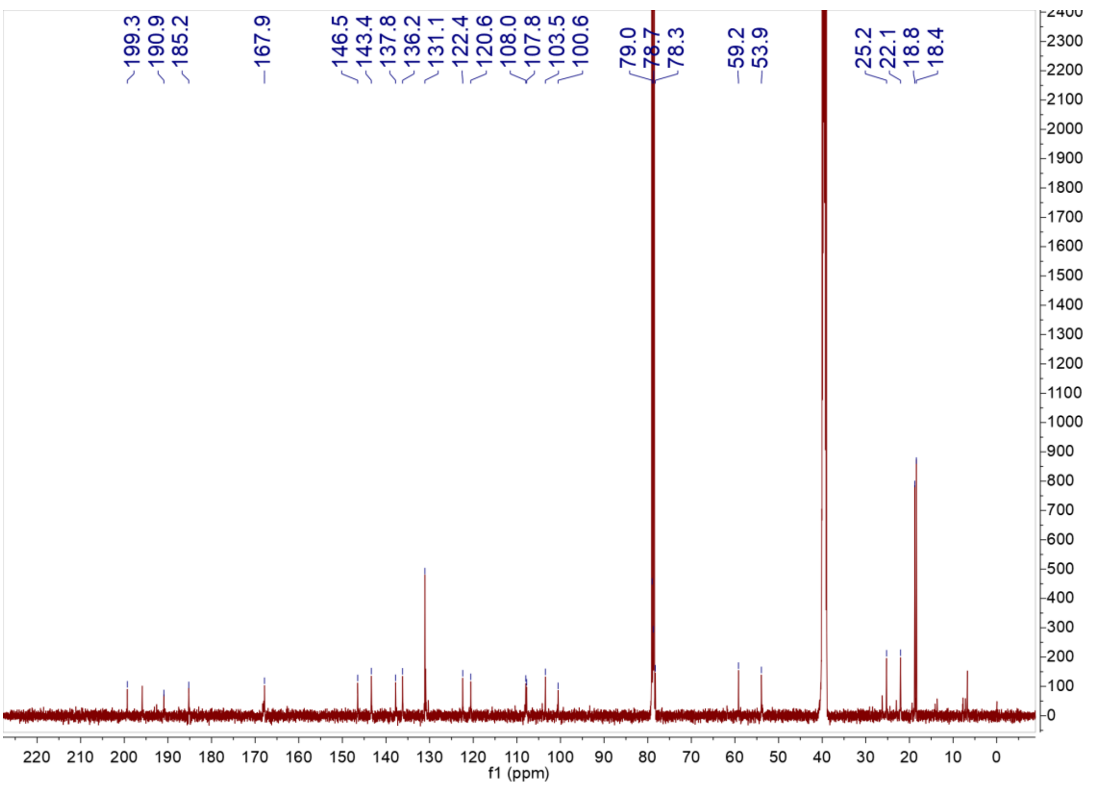

Figure S14. ^{13}C NMR spectrum of acresorbicillinol C (**3**; 125 MHz, DMSO:$CDCl_3$ = 3:1)

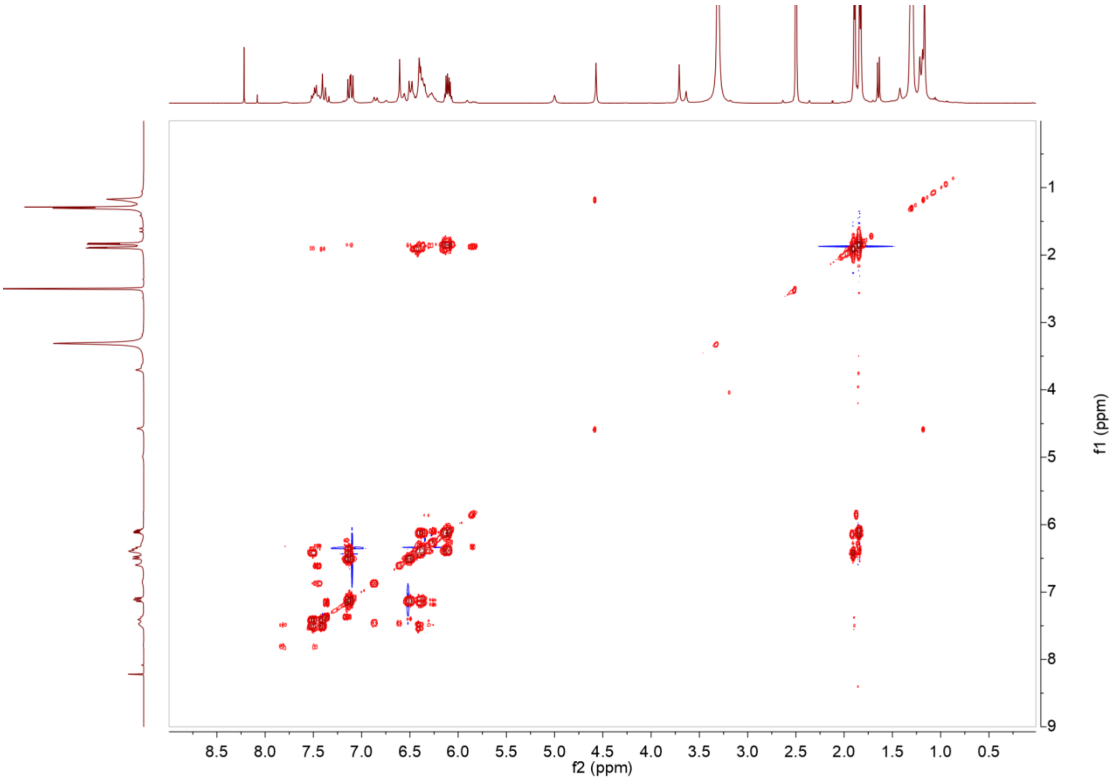

Figure S15. ^{1}H-^{1}H COSY spectrum of acresorbicillinol C (**3**, DMSO:$CDCl_3$ = 3:1)

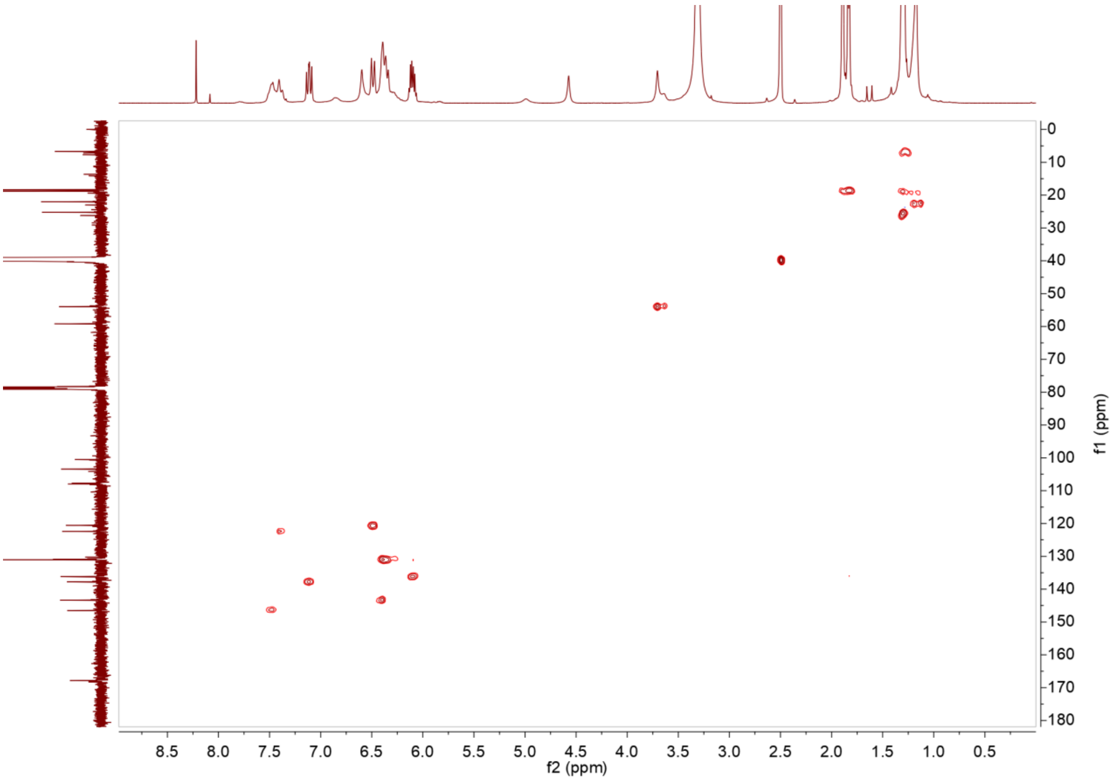

Figure S16. HSQC spectrum of acresorbicillinol C (**3**, DMSO:$CDCl_3$ = 3:1)

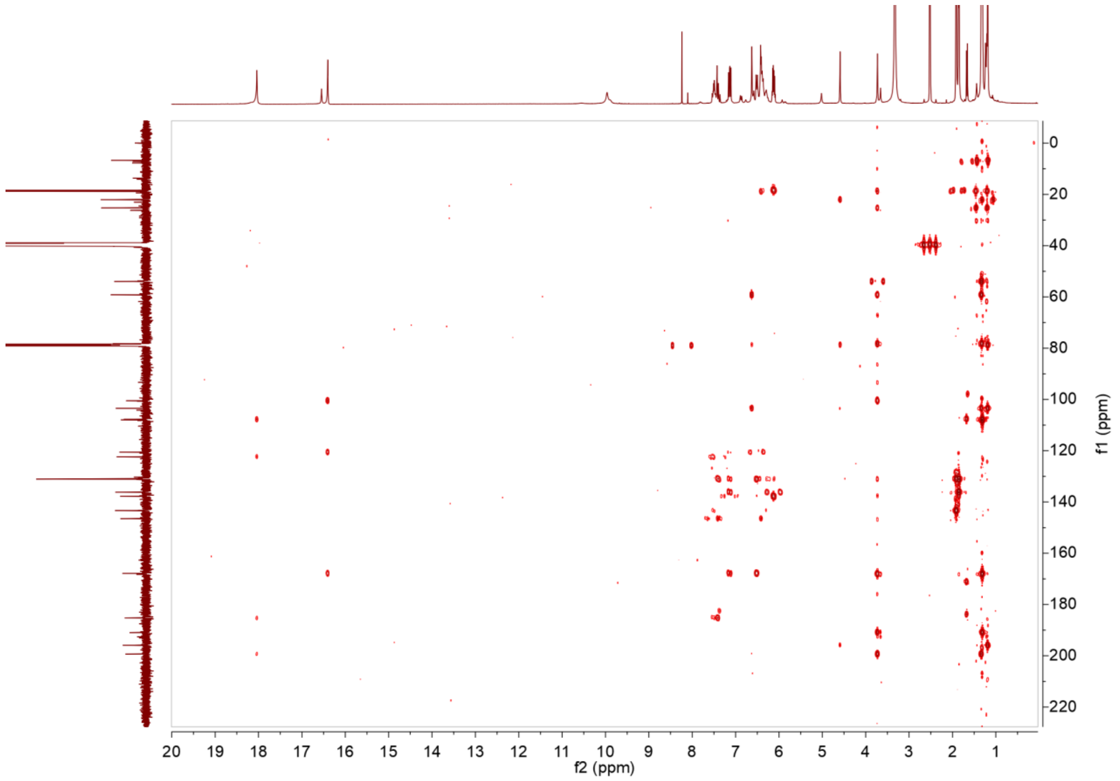

Figure S17. HMBC spectrum of acresorbicillinol C (**3**, DMSO:$CDCl_3$ = 3:1)

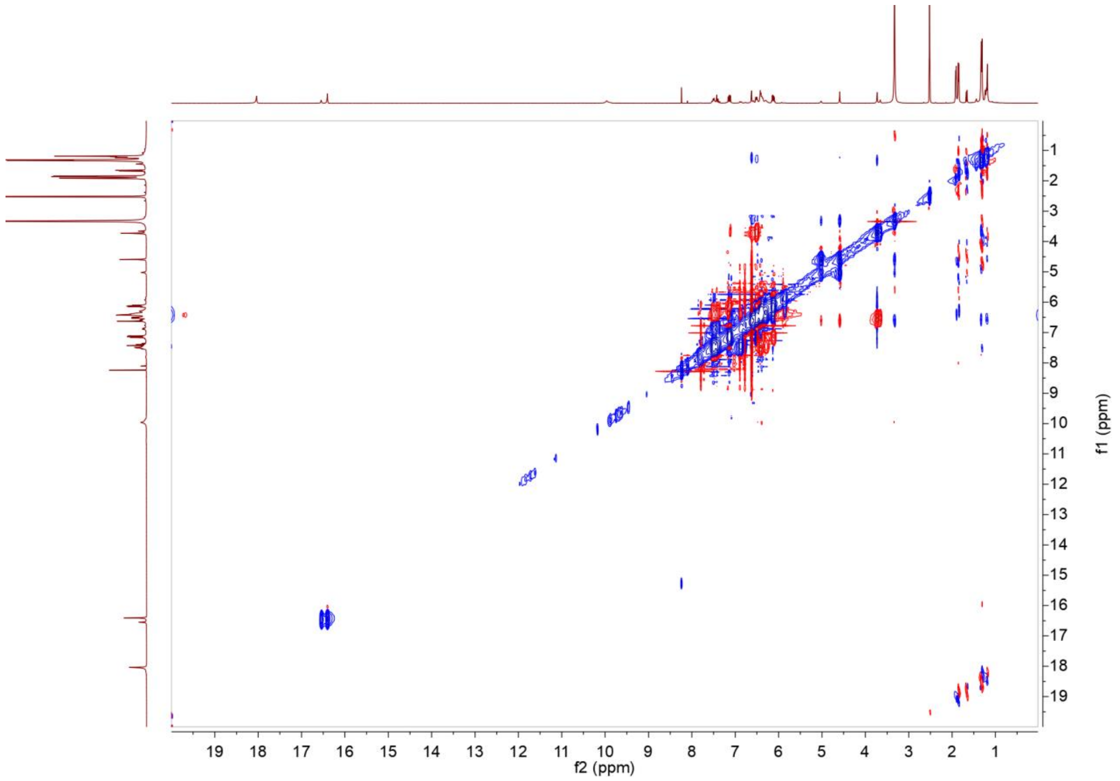

Figure S18. NOESY spectrum of acresorbicillinol C (**3**, DMSO:$CDCl_3$ = 3:1)

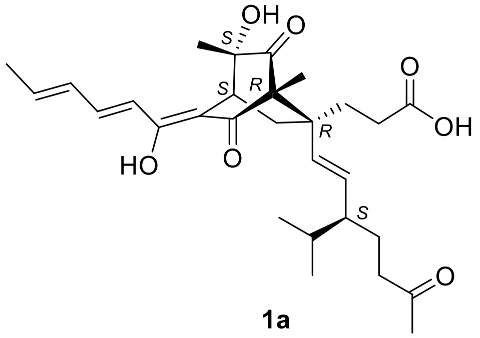

1a

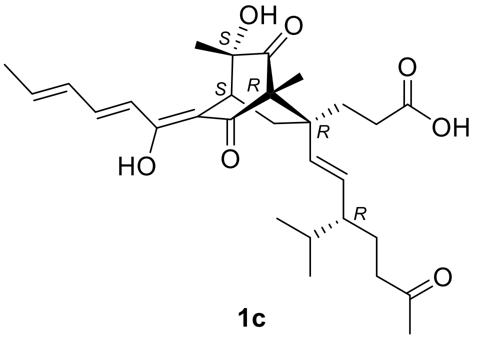

1c

Conformers	Populations	Conformers	Populations
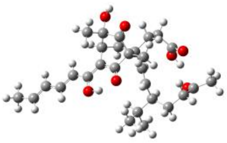	25.49%	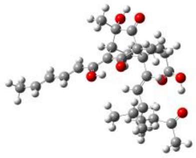	68.26%
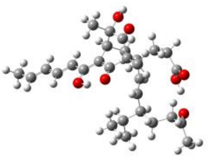	53.89%	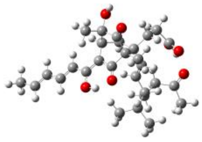	31.74%
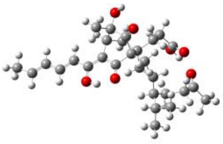	20.62%		

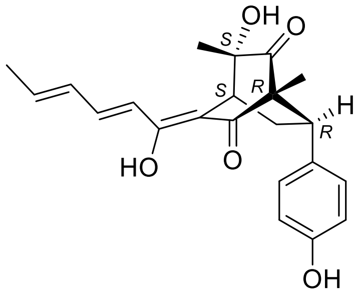

2a

Conformers	Populations
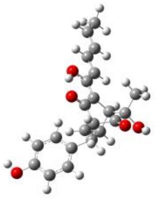	51.44%
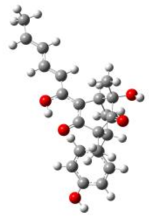	48.56%

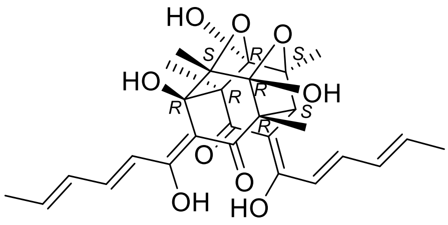

3a

Conformers	Populations
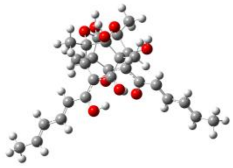	1.97%
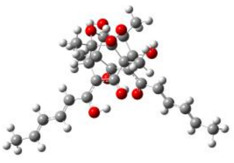	8.24%

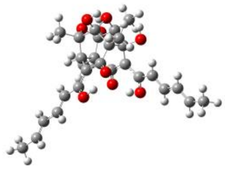	3.15%
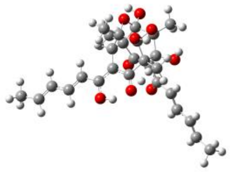	12.10%
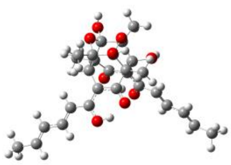	15.53%
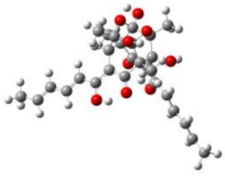	53.82%
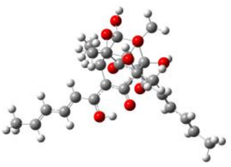	1.01%

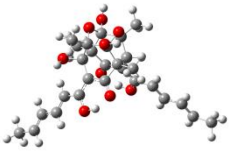	4.17%

Figure S19. ECD conformers of acresorbicillinols A–C (**1**–**3**)

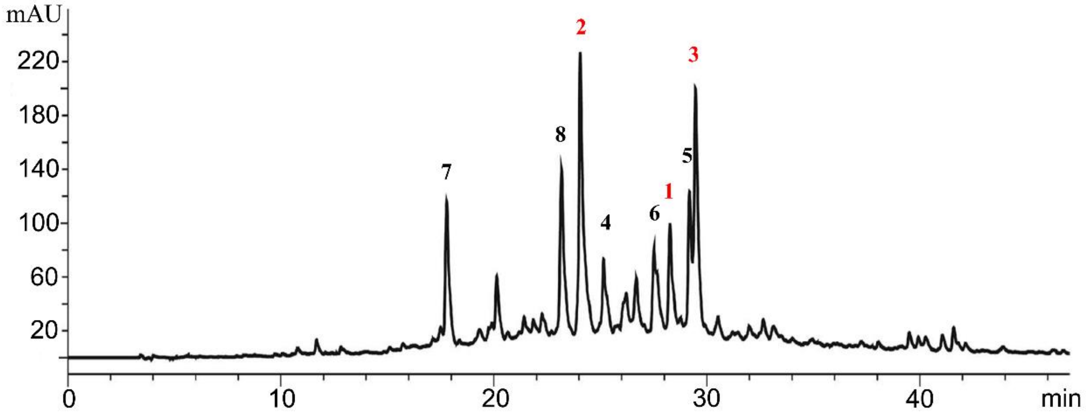

Figure S20. HPLC profiles of the extracts from the rice solid medium of *A. chrysogenum* after 7 days fermentation

Table S1. Primers used in this study

Primer	Sequence
actin-F(RT)	AGTCCAAGCGTGGTATCC
actin-R(RT)	TAGAAGGCAGGGGCGTTG
AcsorA-F(RT)	CGCACTTCTTTGACCTCCG
AcsorA-R(RT)	TTGGCACCGCCCACGAT
AcsorB-F(RT)	GCCATCCTGGGACCACT
AcsorB-R(RT)	TGAACACGCCGAGACAAC
AcsorC-F(RT)	TCGGGGAAGGGTACAAGGC
AcsorC-R(RT)	CTCGACGGACCGCAAGTT
AcsorD-F(RT)	TGATGGCAATTCCGTCTGG
AcsorD-R(RT)	GACTCGTTGAGGGCGTTCC
AcsorE-F(RT)	GGGCTCGTTCCGTCTCGTCT
AcsorE-R(RT)	CCTCGGGCTGGGATTTGAT
AcsorT-F(RT)	GTATTCTCGGCGTCGTCTTC
AcsorT-R(RT)	TTCTTCTTGCCCTGGTTCTT
AcsorR1-F(RT)	CGATGTGGACGACTTGAGG
AcsorR1-R(RT)	TCATACTGCGAGTCCTTGTTAG
AcsorR2-F(RT)	GCTGACCAGTACCGCATCG
AcsorR2-R(RT)	GCCCGCCCTAACGTCTAT
orf1-F(RT)	CGGGATTGTTTCCAAGTCG
orf1-R(RT)	TCCACCAGGGTTGTGCC
orf2-F(RT)	CGGAGATACAGGAAGGC
orf2-R(RT)	CCGCTAAGGCTAGGGTC
